# Supplementary material for: Synthesis and Structure of Nido-Carboranyl Azide and Its “Click” Reactions
Source: Molecules. 2021 Jan 20;26(3):530. doi: 10.3390/molecules26030530 (PMC7930967; doi:10.3390/molecules26030530)
Supplement: Supplementary file 1 [file molecules-26-00530-s001.zip › Supporting Information.pdf]

SUPPORTING INFORMATION

Synthesis and structure of *nido*-carboranyl azide and its "click" reactions

Anna A. Druzina,* Olga B. Zhidkova, Nadezhda V. Dudarova, Irina D. Kosenko, Ivan V. Ananyev, Sergey V. Timofeev and Vladimir I. Bregadze

A.N. Nesmeyanov Institute of Organoelement Compounds, Russian Academy of Sciences, 28 Vavilov Str., 119991 Moscow, Russia; Zolga57@mail.ru (O.B.Z.); nadezjdino_96@mail.ru (N.V.D.); kosenko@ineos.ac.ru (I.D.K.); i.ananyev@gmail.com (I.V.A.); timofeev@ineos.ac.ru (S.V.T.); bre@ineos.ac.ru (V.I.B).

* Correspondence: ilinova_anna@mail.ru; Tel.: +7-926-404-5566 (A.A.D.)

^{1}H, ^{11}B and ^{13}C NMR, IR and high-resolution mass spectra of compounds **2,3,5,10-12**

Display Report

Analysis Info

Analysis Name	D:\Data\Chizhov\INEOS\Laskova\da-030_&clblow.d	Acquisition Date	29.09.2020 13:58:17
Method	tune_low_1550.m	Operator	BDAL@DE
Sample Name	/CHIZ DA-030	Instrument	maXis 43
Comment	CH3CN !00 %, dil. 20, calibrant added		

Acquisition Parameter

Source Type	ESI	Ion Polarity	Positive	Set Nebulizer	0.4 Bar
Focus	Not active	Set Capillary	4500 V	Set Dry Heater	180 °C
Scan Begin	50 m/z	Set End Plate Offset	-500 V	Set Dry Gas	4.0 l/min
Scan End	1550 m/z	Set Collision Cell RF	300.0 Vpp	Set Divert Valve	Source

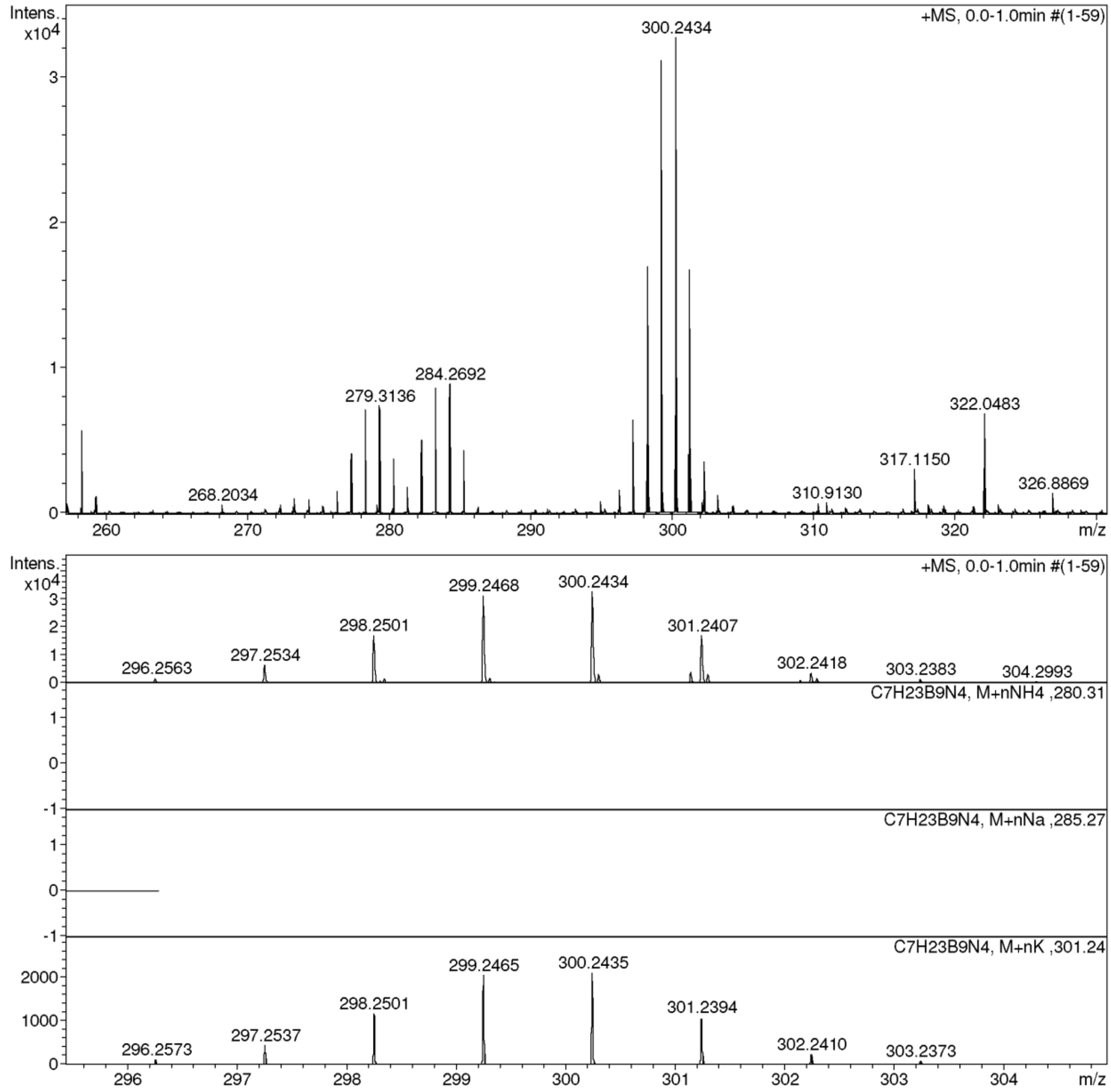

Figure S1. ESI-HRMS spectrum of compound **2**

Display Report

Analysis Info

Analysis Name	D:\Data\Chizhov\INEOS\Laskova\da-035_&clb.d	Acquisition Date	29.09.2020 14:26:58
Method	tune_wide.m	Operator	BDAL@DE
Sample Name	/CHIZ DA-035	Instrument	maXis 43
Comment	CH3CN !00 %, dil. 200, calibrant added		

Acquisition Parameter

Source Type	ESI	Ion Polarity	Positive	Set Nebulizer	0.5 Bar
Focus	Active	Set Capillary	4500 V	Set Dry Heater	180 °C
Scan Begin	50 m/z	Set End Plate Offset	-500 V	Set Dry Gas	4.0 l/min
Scan End	3000 m/z	Set Collision Cell RF	1200.0 Vpp	Set Divert Valve	Waste

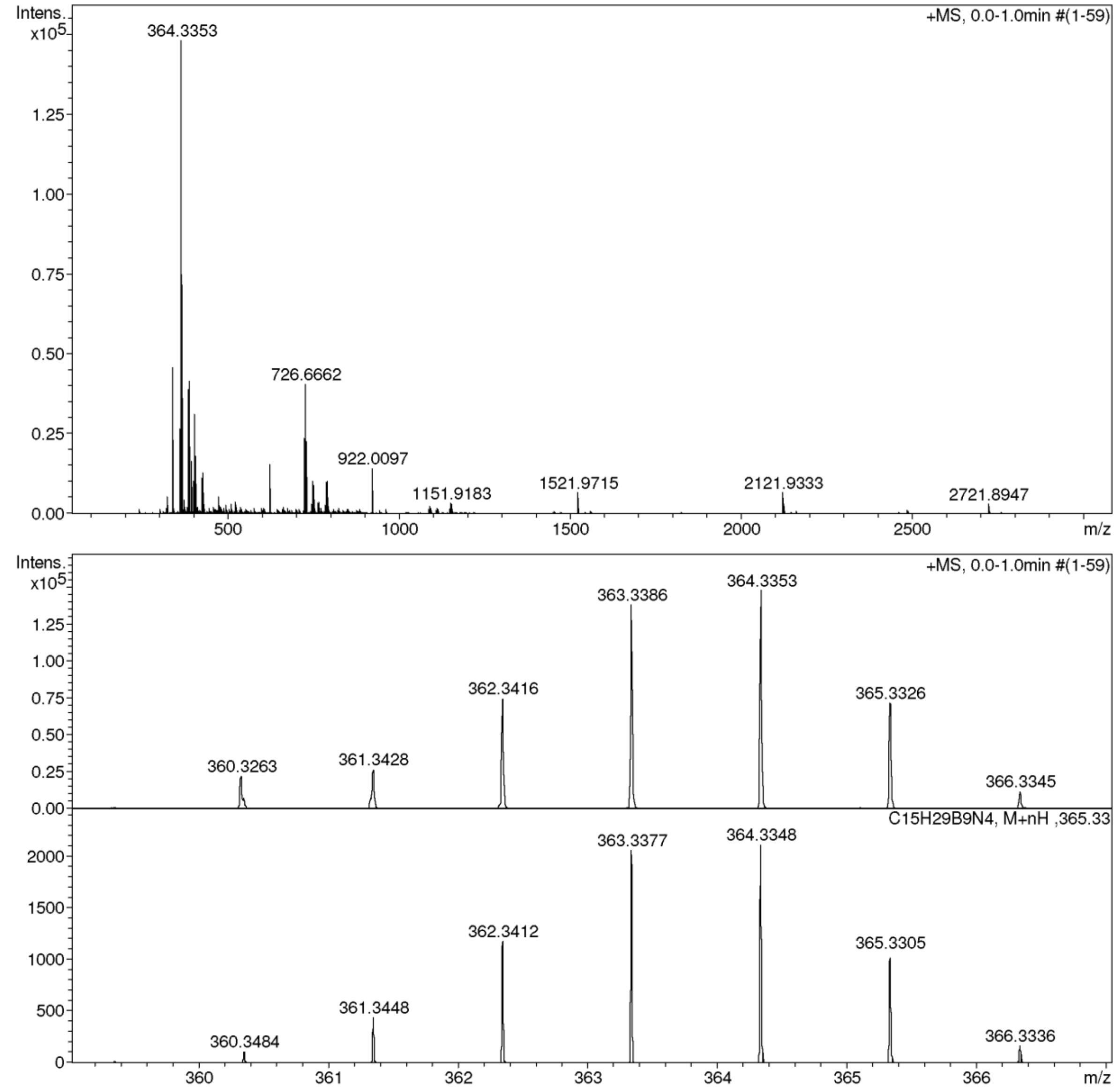

Figure S2. ESI-HRMS spectrum of compound **3**

Display Report

Analysis Info

Analysis Name	D:\Data\Chizhov\INEOS\Laskova\da-037_&clb.d	Acquisition Date	29.09.2020 14:35:42
Method	tune_wide.m	Operator	BDAL@DE
Sample Name	/CHIZ DA-037	Instrument	maXis 43
Comment	CH3CN !00 %, dil. 200, calibrant added		

Acquisition Parameter

Source Type	ESI	Ion Polarity	Positive	Set Nebulizer	0.5 Bar
Focus	Active	Set Capillary	4500 V	Set Dry Heater	180 °C
Scan Begin	50 m/z	Set End Plate Offset	-500 V	Set Dry Gas	4.0 l/min
Scan End	3000 m/z	Set Collision Cell RF	1200.0 Vpp	Set Divert Valve	Waste

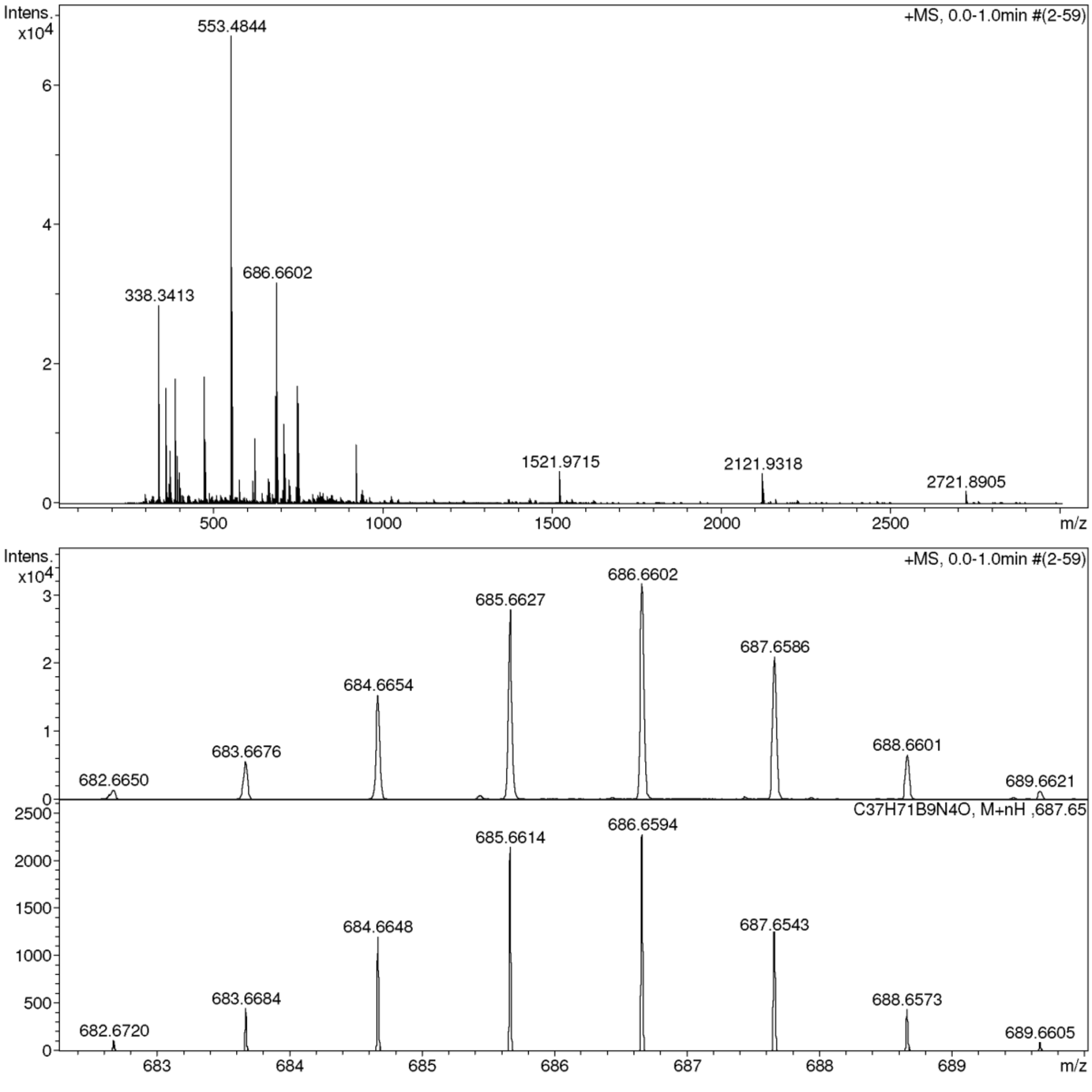

Figure S3. ESI-HRMS spectrum of compound **5**

Display Report

Analysis Info

Analysis Name	D:\Data\Chizhov\INEOS\Laskova\da-032_&clb.d	Acquisition Date	29.09.2020 14:11:16
Method	tune_wide.m	Operator	BDAL@DE
Sample Name	/CHIZ DA-032	Instrument	maXis 43
Comment	CH3CN !00 %, dil. 20, calibrant added		

Acquisition Parameter

Source Type	ESI	Ion Polarity	Positive	Set Nebulizer	0.5 Bar
Focus	Active	Set Capillary	4500 V	Set Dry Heater	180 °C
Scan Begin	50 m/z	Set End Plate Offset	-500 V	Set Dry Gas	4.0 l/min
Scan End	3000 m/z	Set Collision Cell RF	1200.0 Vpp	Set Divert Valve	Waste

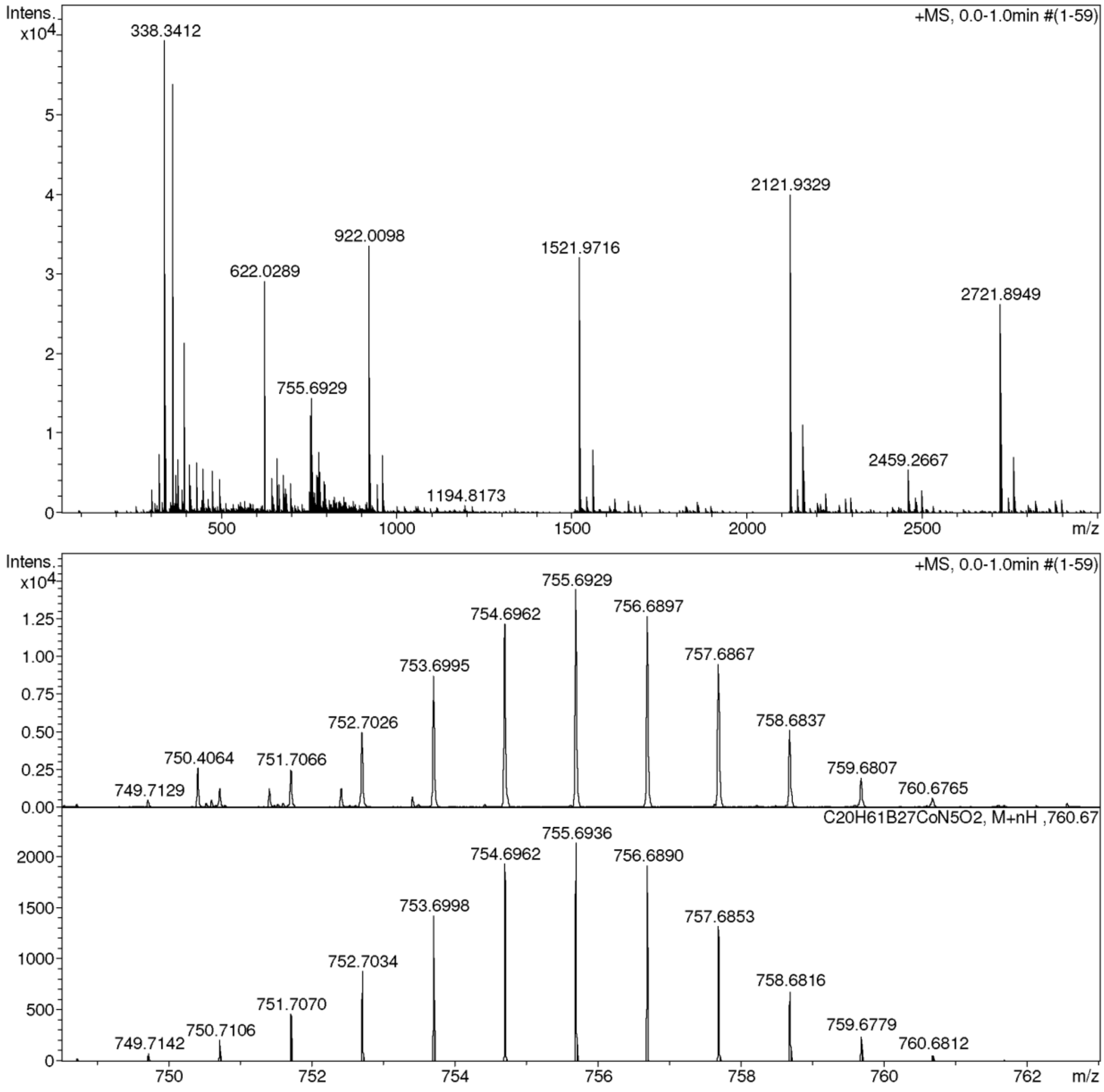

Bruker Compass DataAnalysis 4.0 printed: 29.09.2020 14:49:23 Page 1 of 1

Figure S4. ESI-HRMS spectrum of compound **10**

Display Report

Analysis Info

Analysis Name	D:\Data\Chizhov\INEOS\Laskova\da-033_&clb.d	Acquisition Date	29.09.2020 14:15:00
Method	tune_wide.m	Operator	BDAL@DE
Sample Name	/CHIZ DA-033	Instrument	maXis 43
Comment	CH3CN !00 %, dil. 20, calibrant added		

Acquisition Parameter

Source Type	ESI	Ion Polarity	Positive	Set Nebulizer	0.5 Bar
Focus	Active	Set Capillary	4500 V	Set Dry Heater	180 °C
Scan Begin	50 m/z	Set End Plate Offset	-500 V	Set Dry Gas	4.0 l/min
Scan End	3000 m/z	Set Collision Cell RF	1200.0 Vpp	Set Divert Valve	Waste

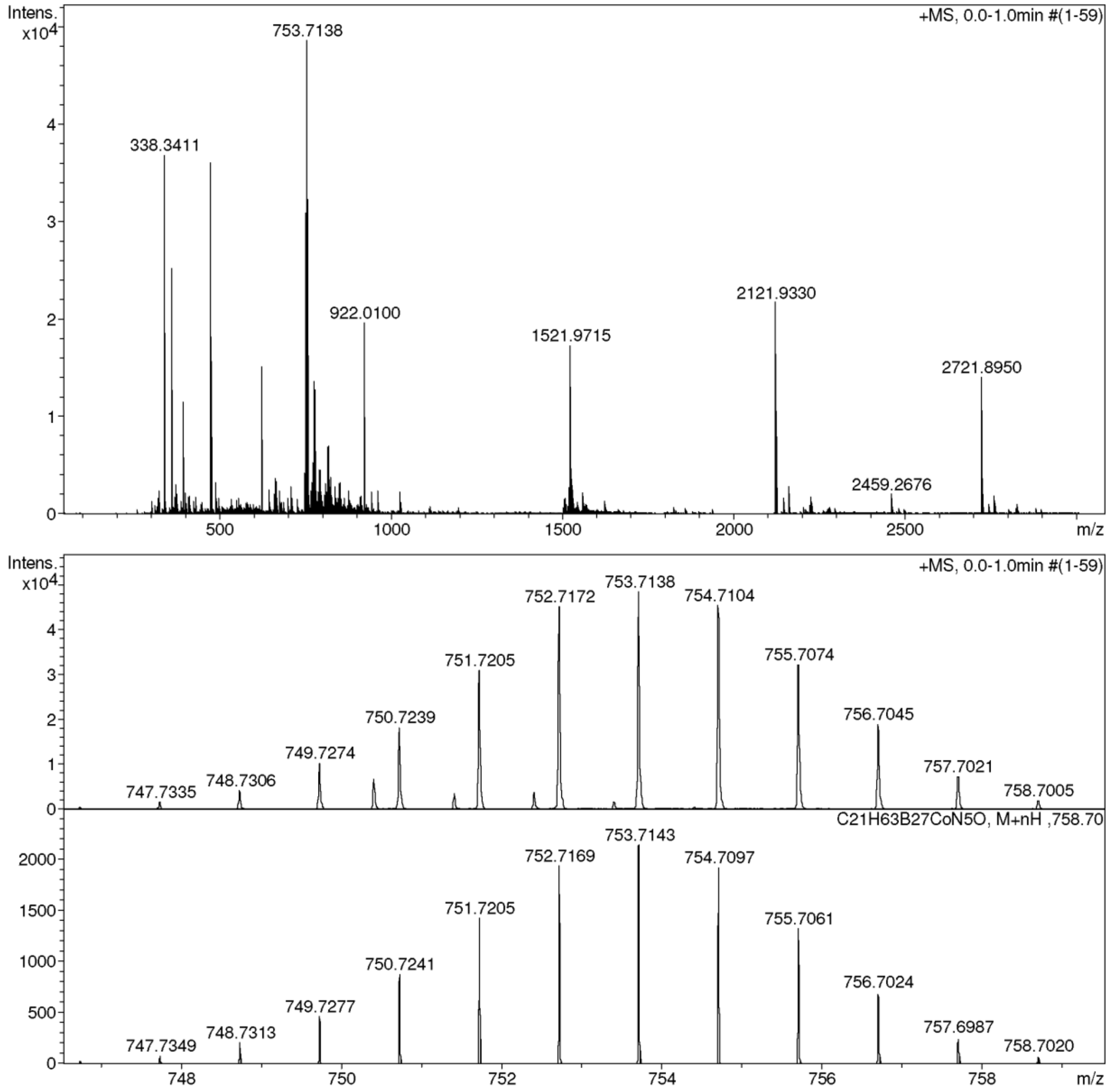

Figure S5. ESI-HRMS spectrum of compound **11**

Display Report

Analysis Info

Analysis Name	D:\Data\Chizhov\INEOS\Laskova\da-034_&clb.d	Acquisition Date	29.09.2020 14:22:18
Method	tune_wide.m	Operator	BDAL@DE
Sample Name	/CHIZ DA-034	Instrument	maXis 43
Comment	CH3CN !00 %, dil. 20, calibrant added		

Acquisition Parameter

Source Type	ESI	Ion Polarity	Positive	Set Nebulizer	0.5 Bar
Focus	Active	Set Capillary	4500 V	Set Dry Heater	180 °C
Scan Begin	50 m/z	Set End Plate Offset	-500 V	Set Dry Gas	4.0 l/min
Scan End	3000 m/z	Set Collision Cell RF	1200.0 Vpp	Set Divert Valve	Waste

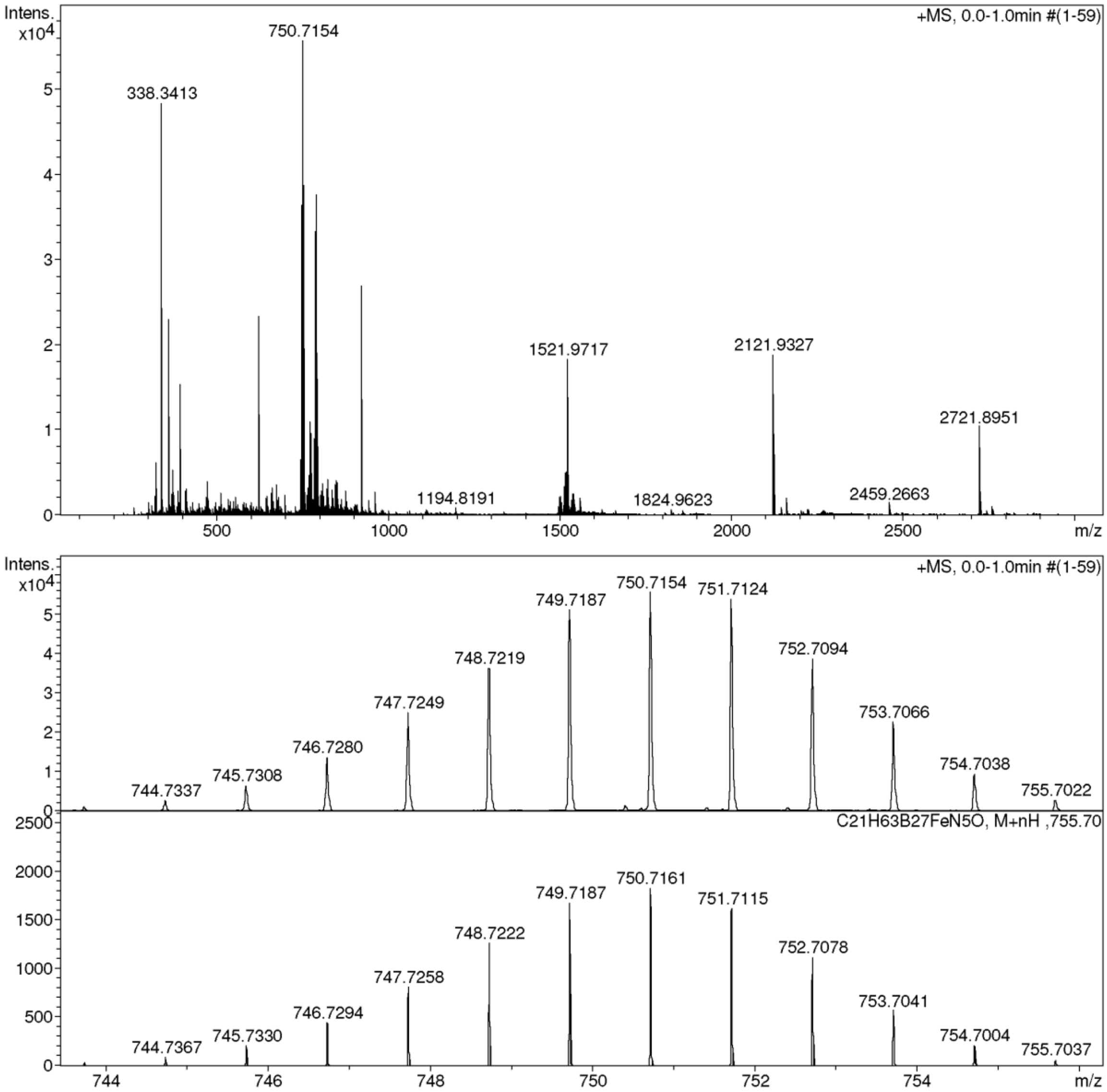

Figure S6. ESI-HRMS spectrum of compound **12**

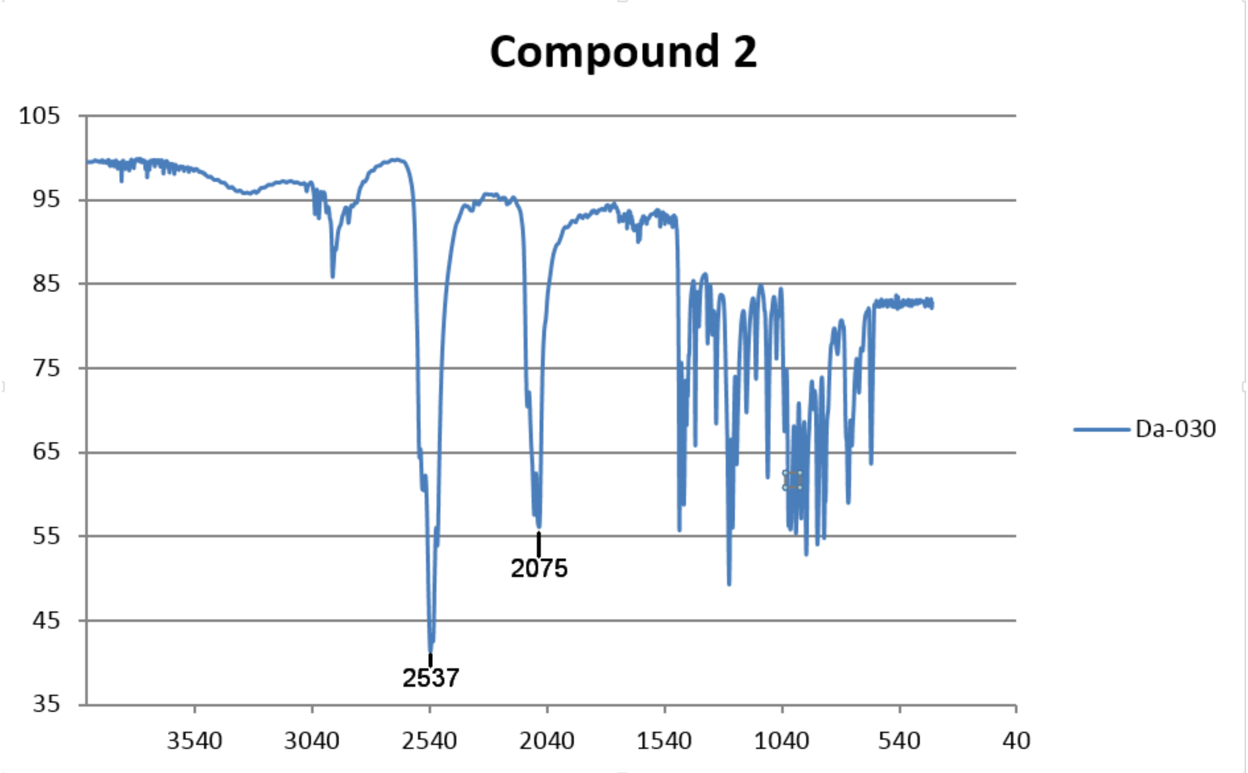

Figure S7. IR spectrum of compound **2**

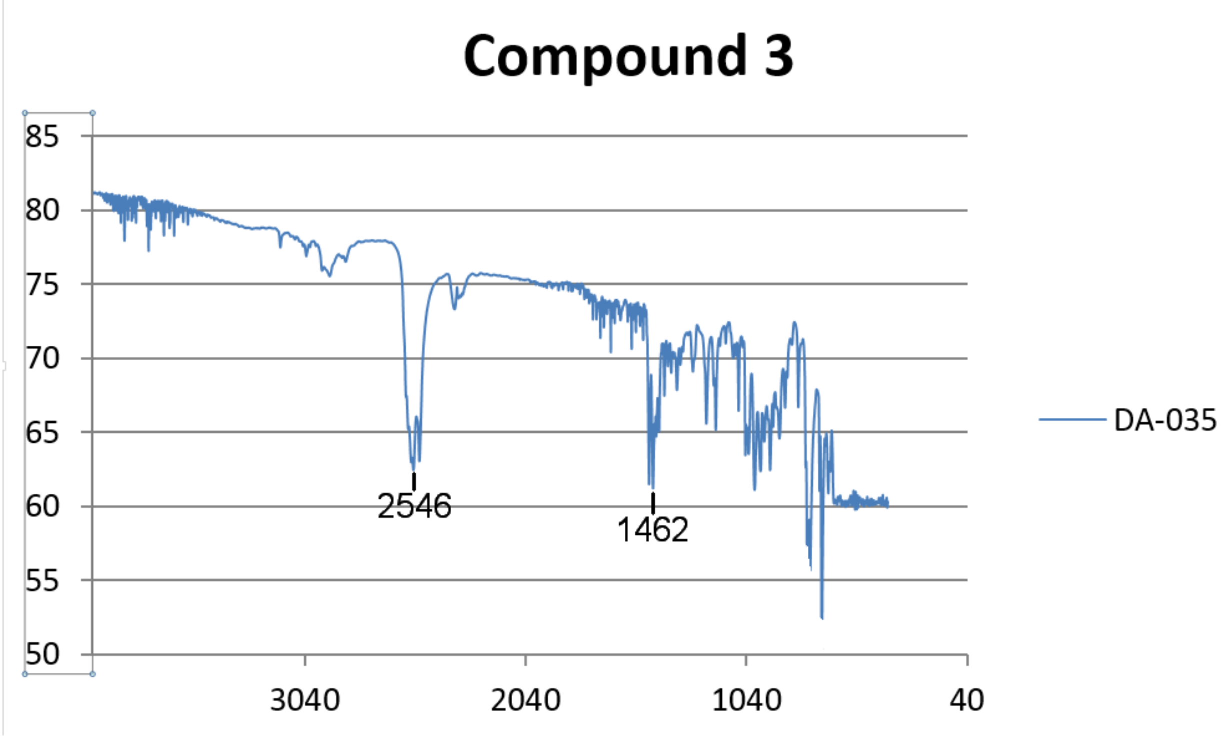

Figure S8. IR spectrum of compound **3**

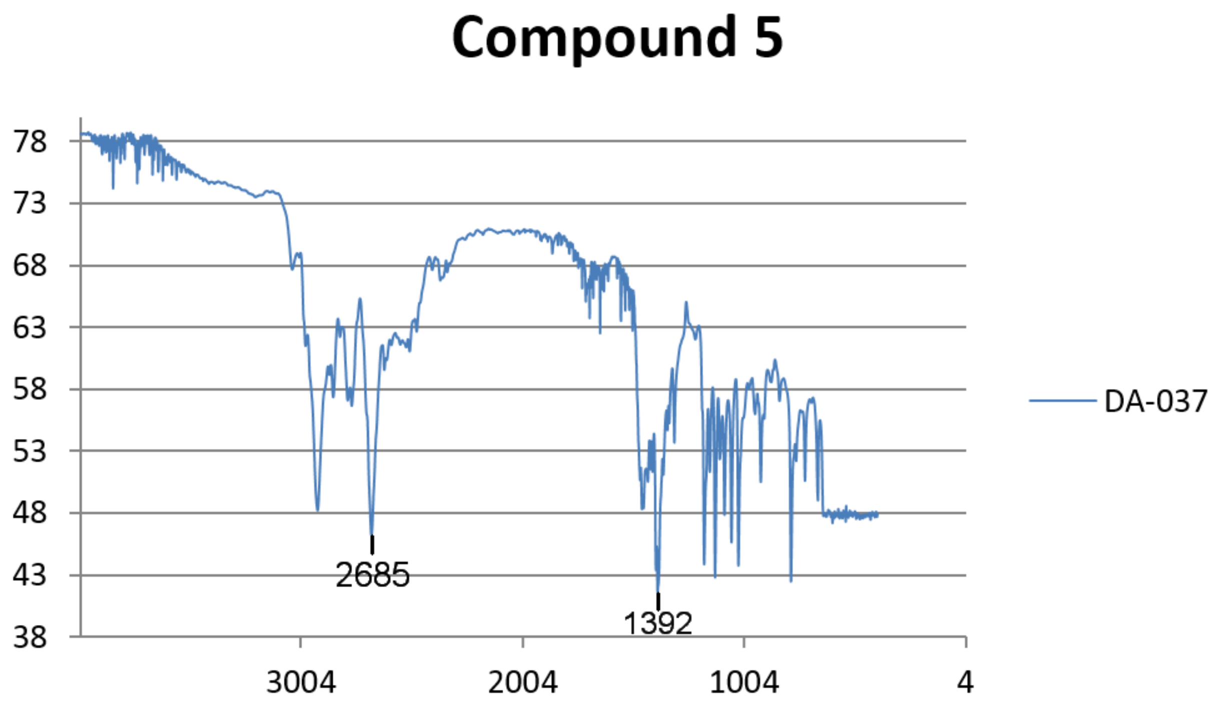

Figure S9. IR spectrum of compound **5**

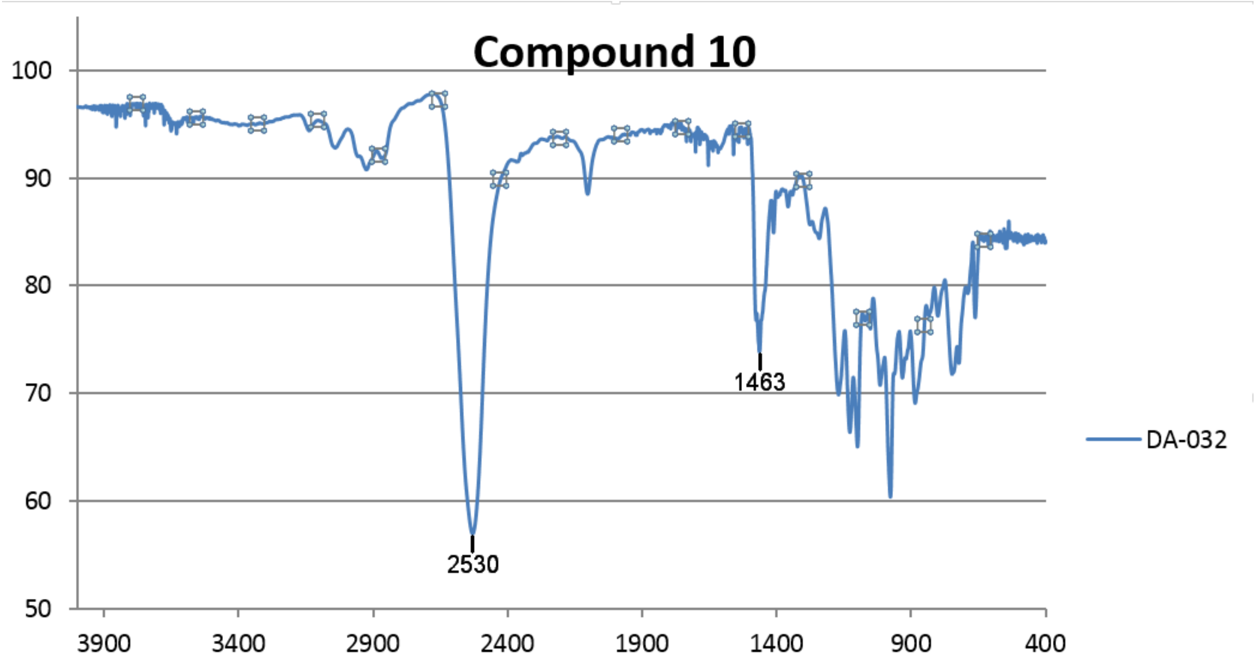

Figure S10. IR spectrum of compound **10**

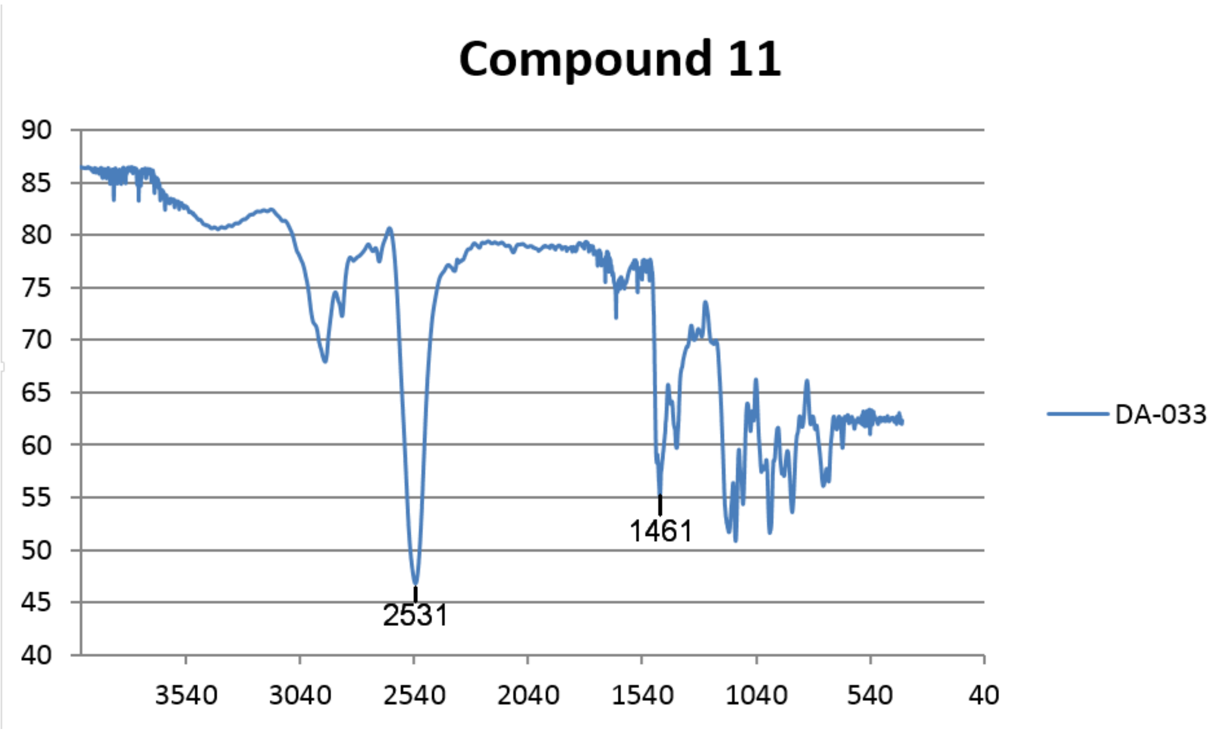

Figure S11. IR spectrum of compound **11**

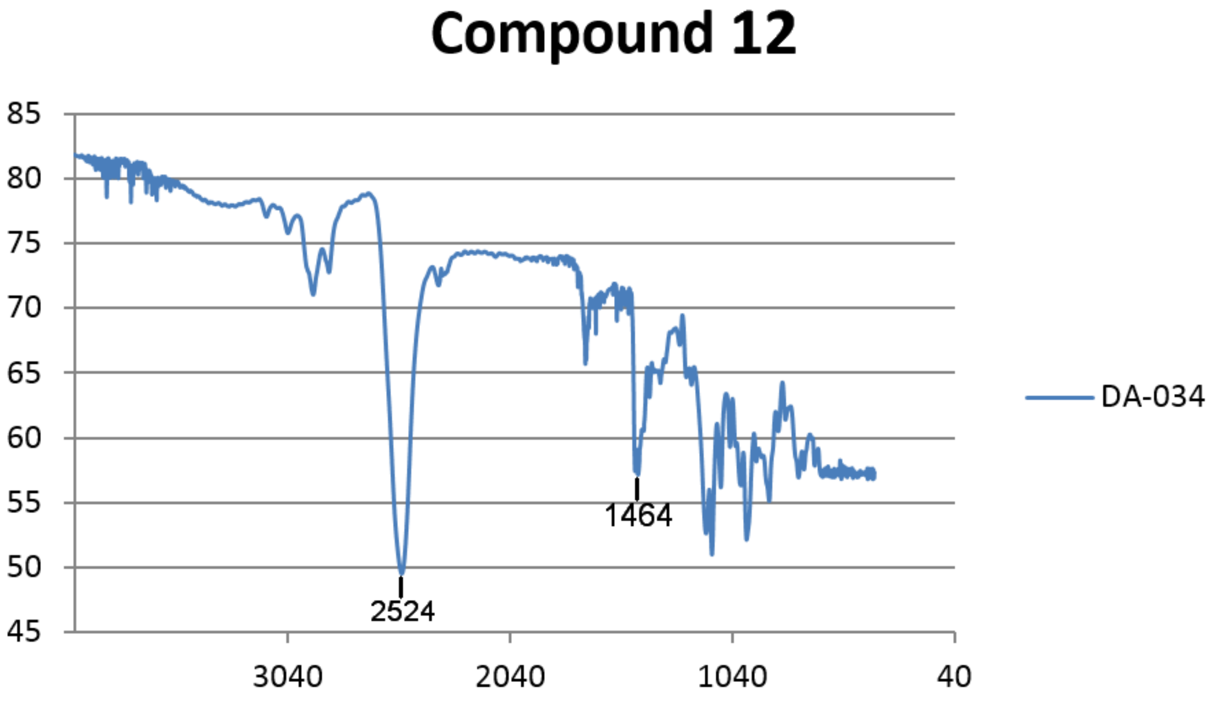

Figure S12. IR spectrum of compound **12**

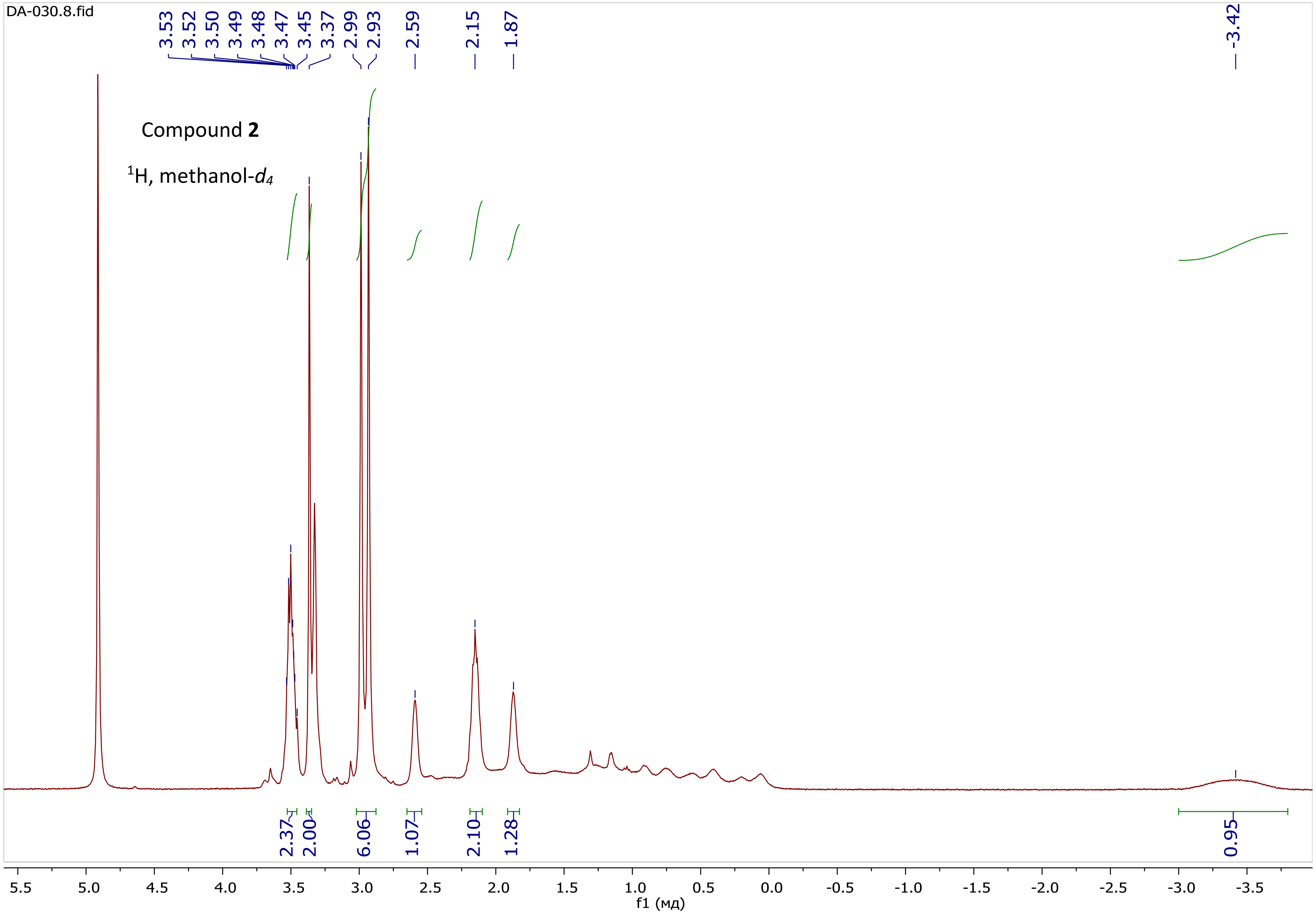

Figure S13. ^{1}H NMR spectrum of compound **2**

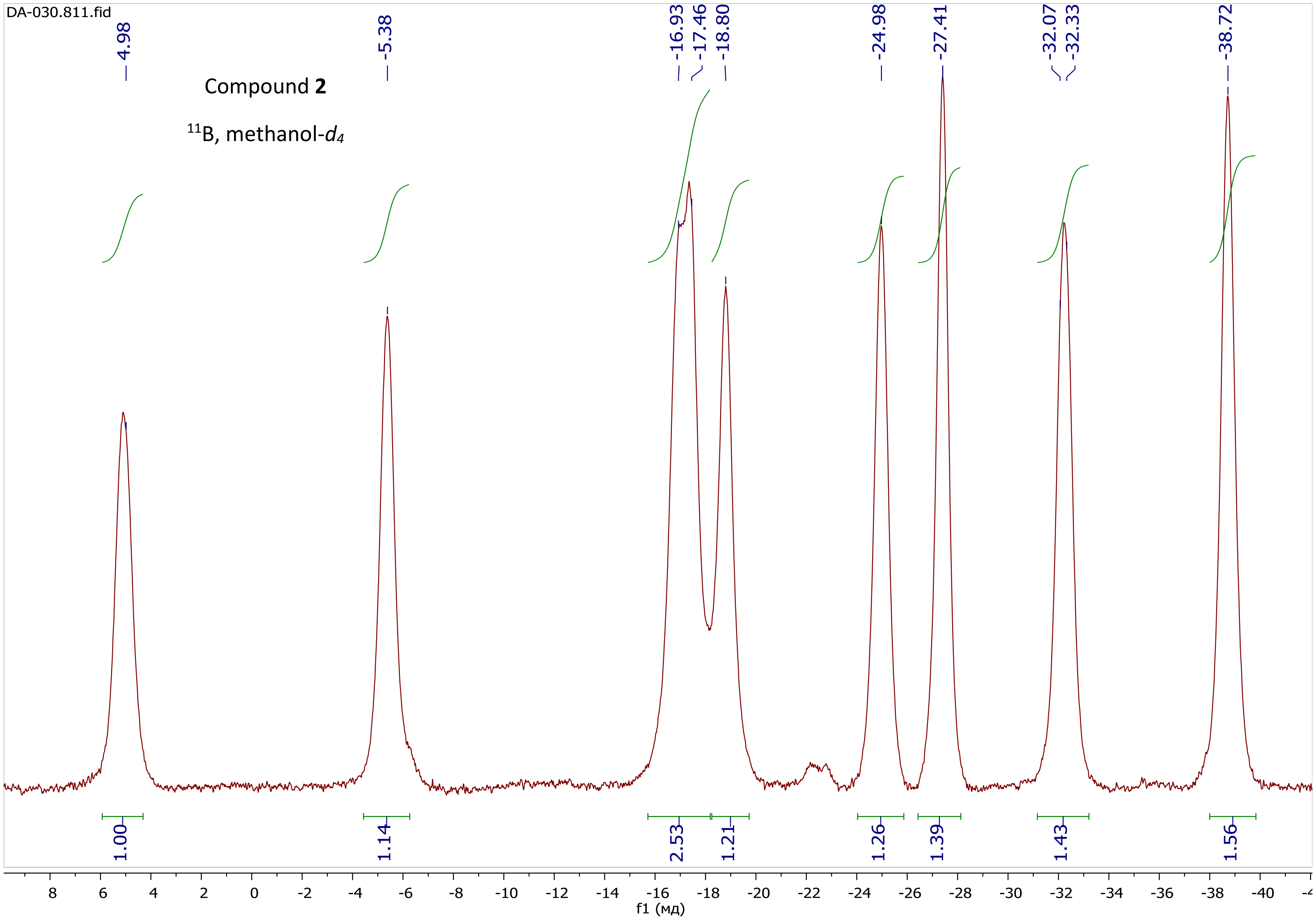

Figure S14. ^{11}B NMR spectrum of compound **2**

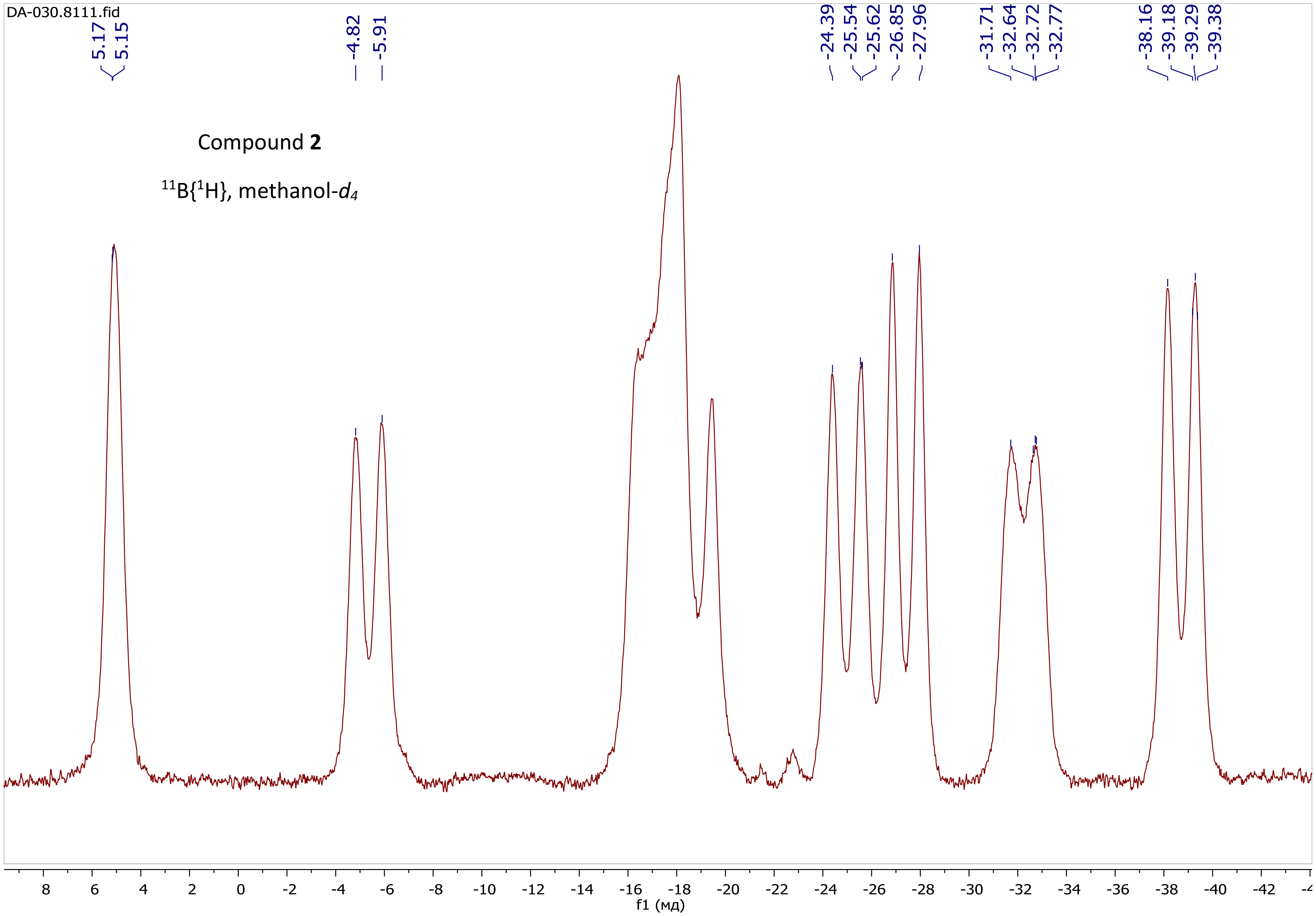

Figure S15. $^{11}B\{^{1}H\}$ NMR spectrum of compound **2**

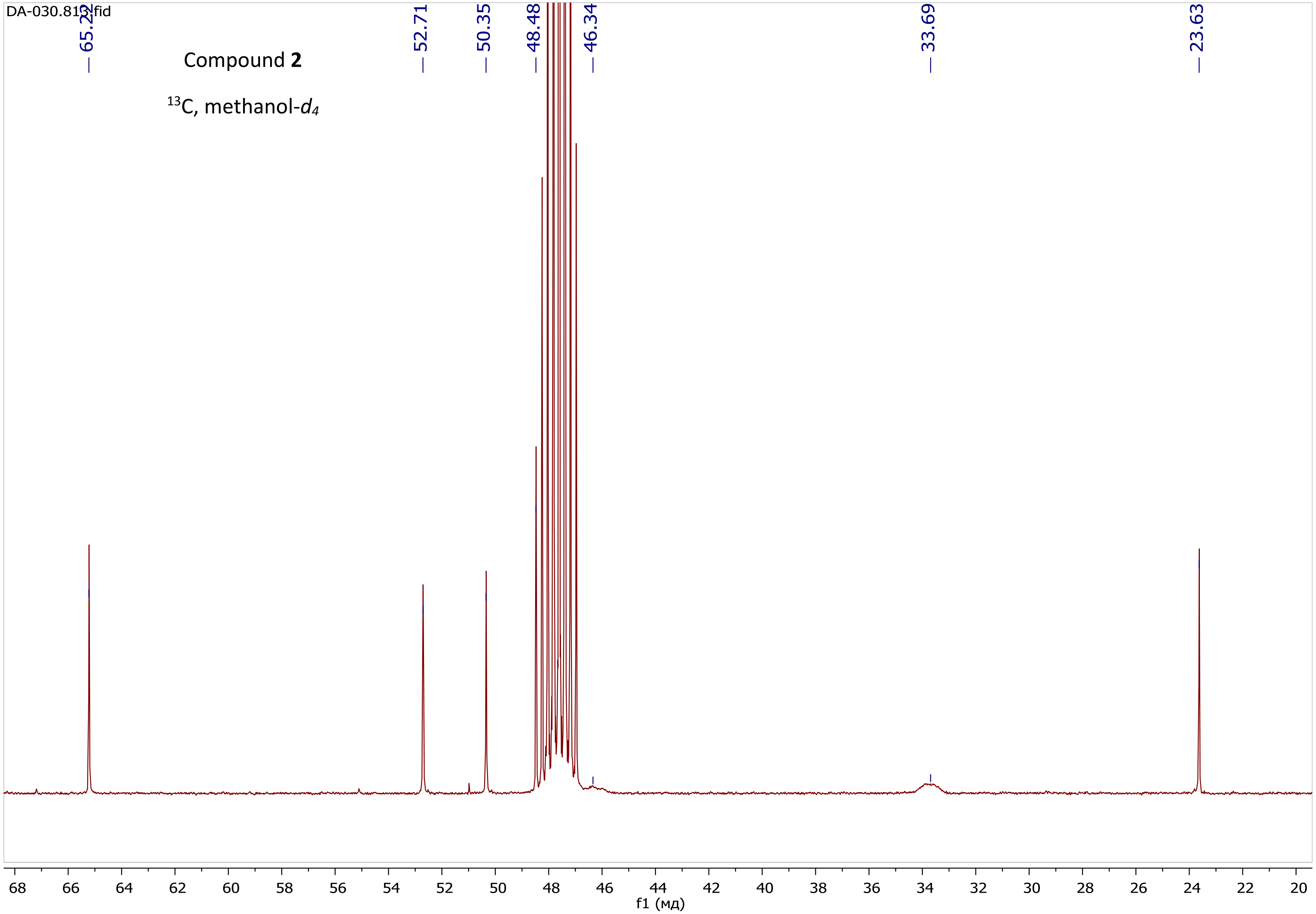

Figure S16. ^{13}C NMR spectrum of compound **2**

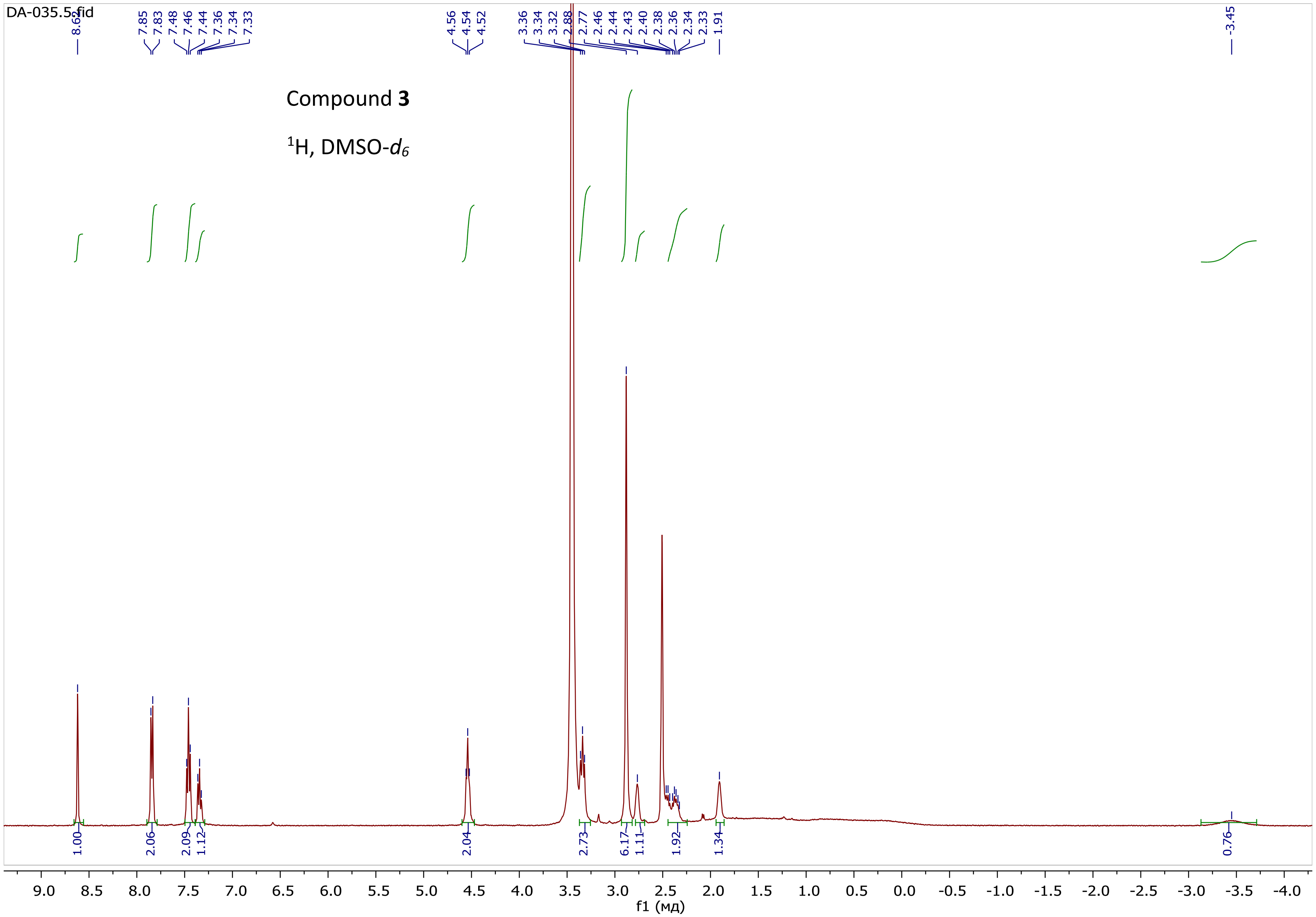

Figure S17. ^{1}H NMR spectrum of compound **3**

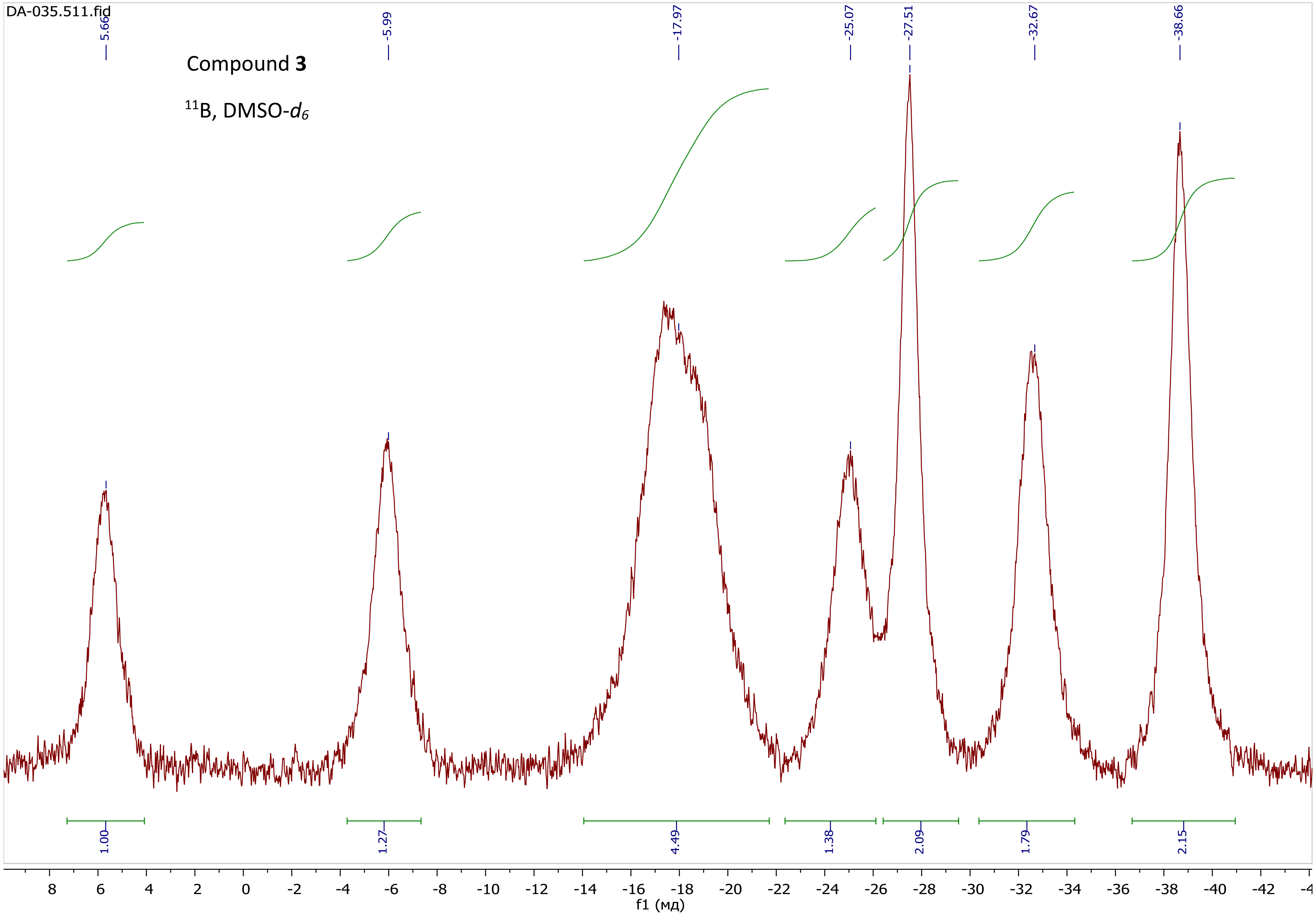

Figure S18. ^{11}B NMR spectrum of compound **3**

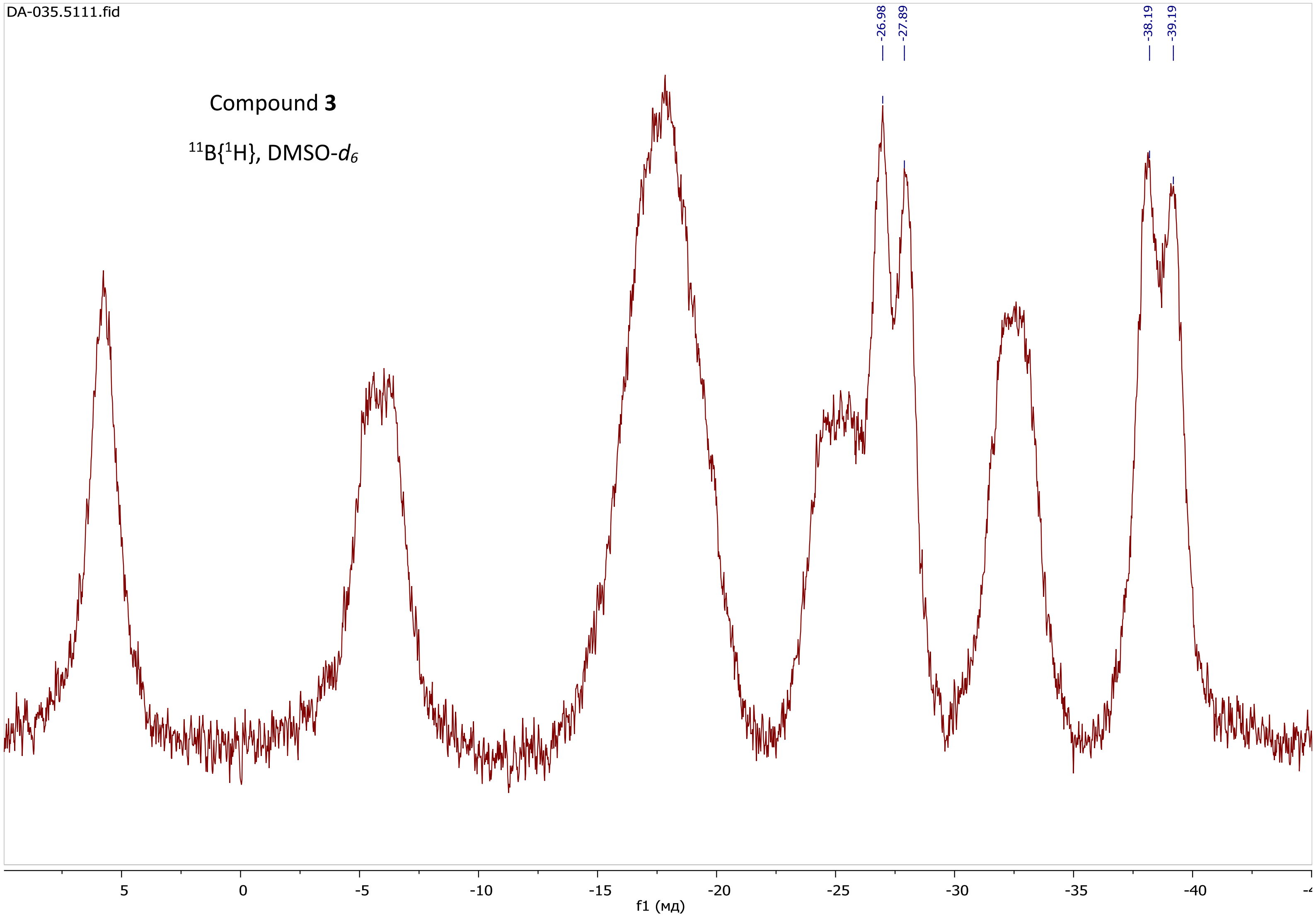

Figure S19. $^{11}B\{^{1}H\}$ NMR spectrum of compound **3**

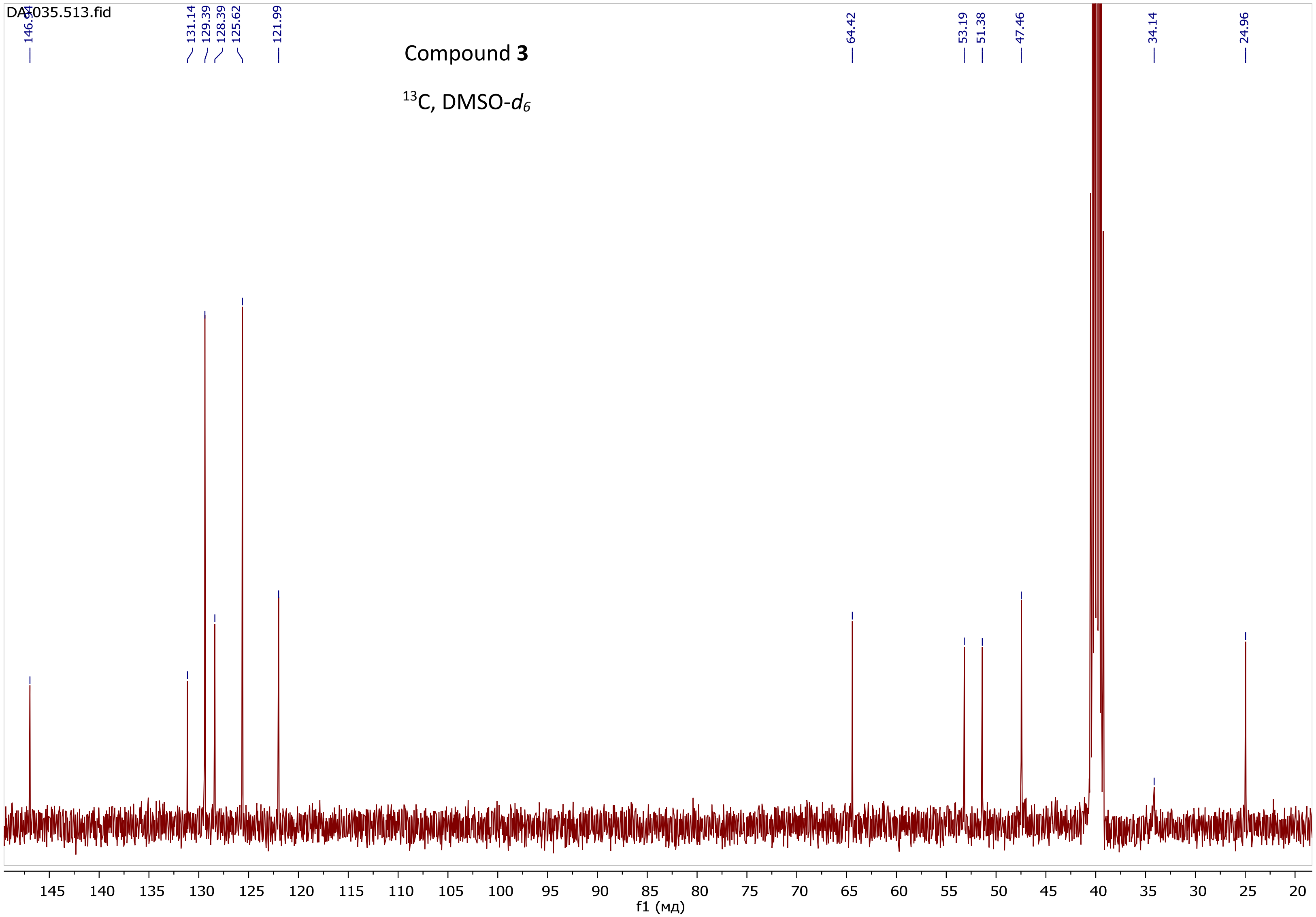

Figure S20. ^{13}C NMR spectrum of compound **3**

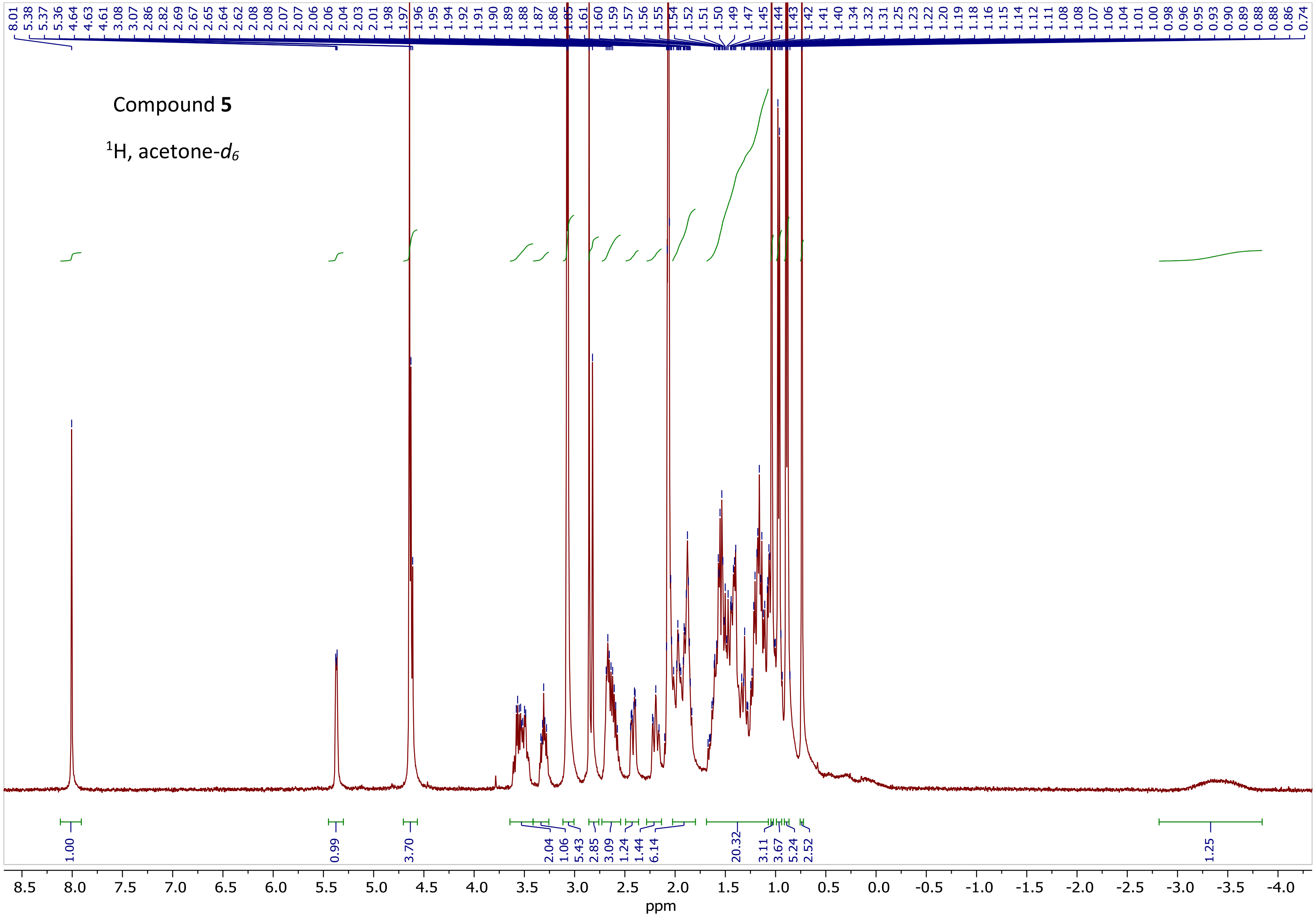

Figure S21. ^{1}H NMR spectrum of compound **5**

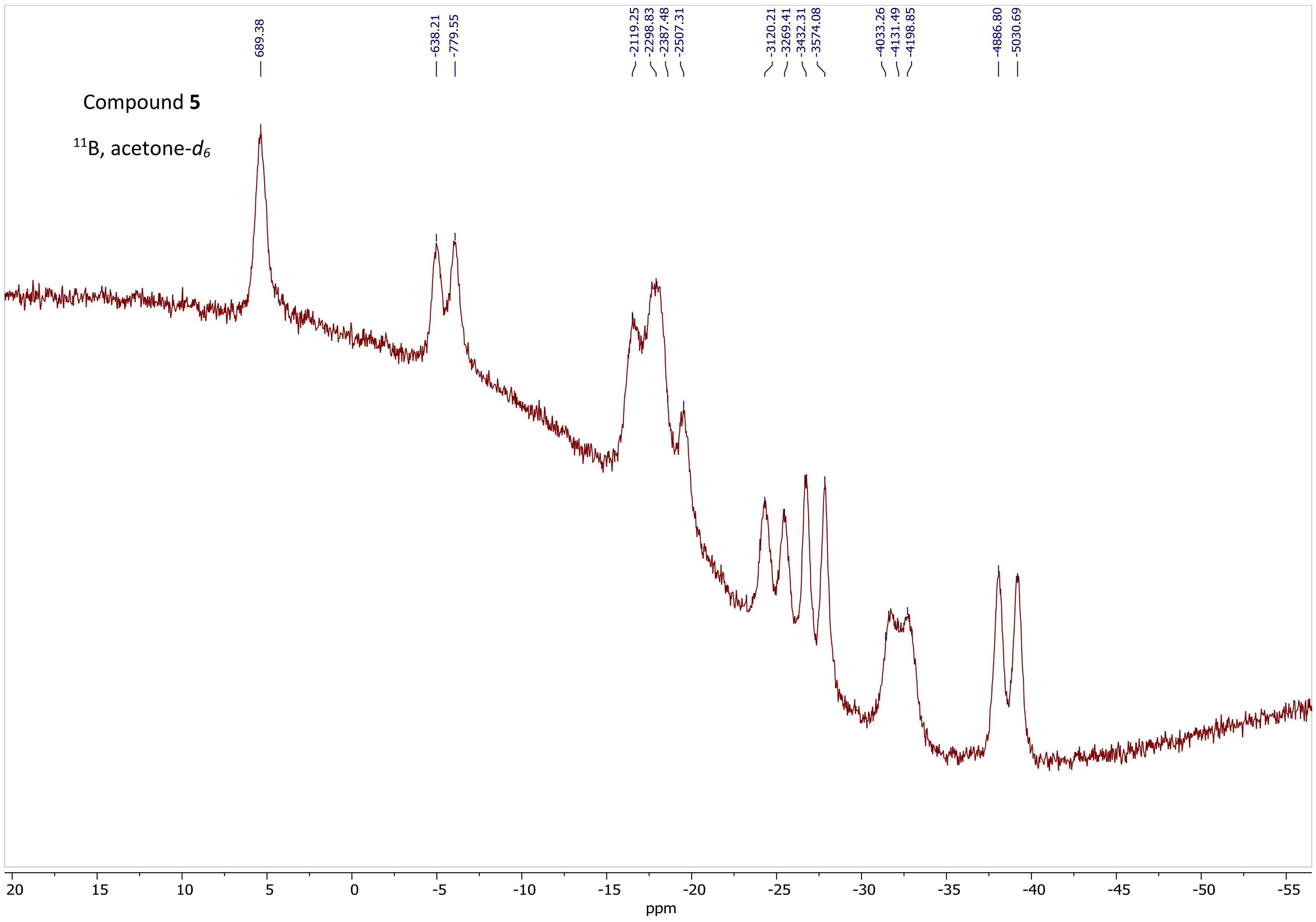

Figure S22. ^{11}B NMR spectrum of compound **5**

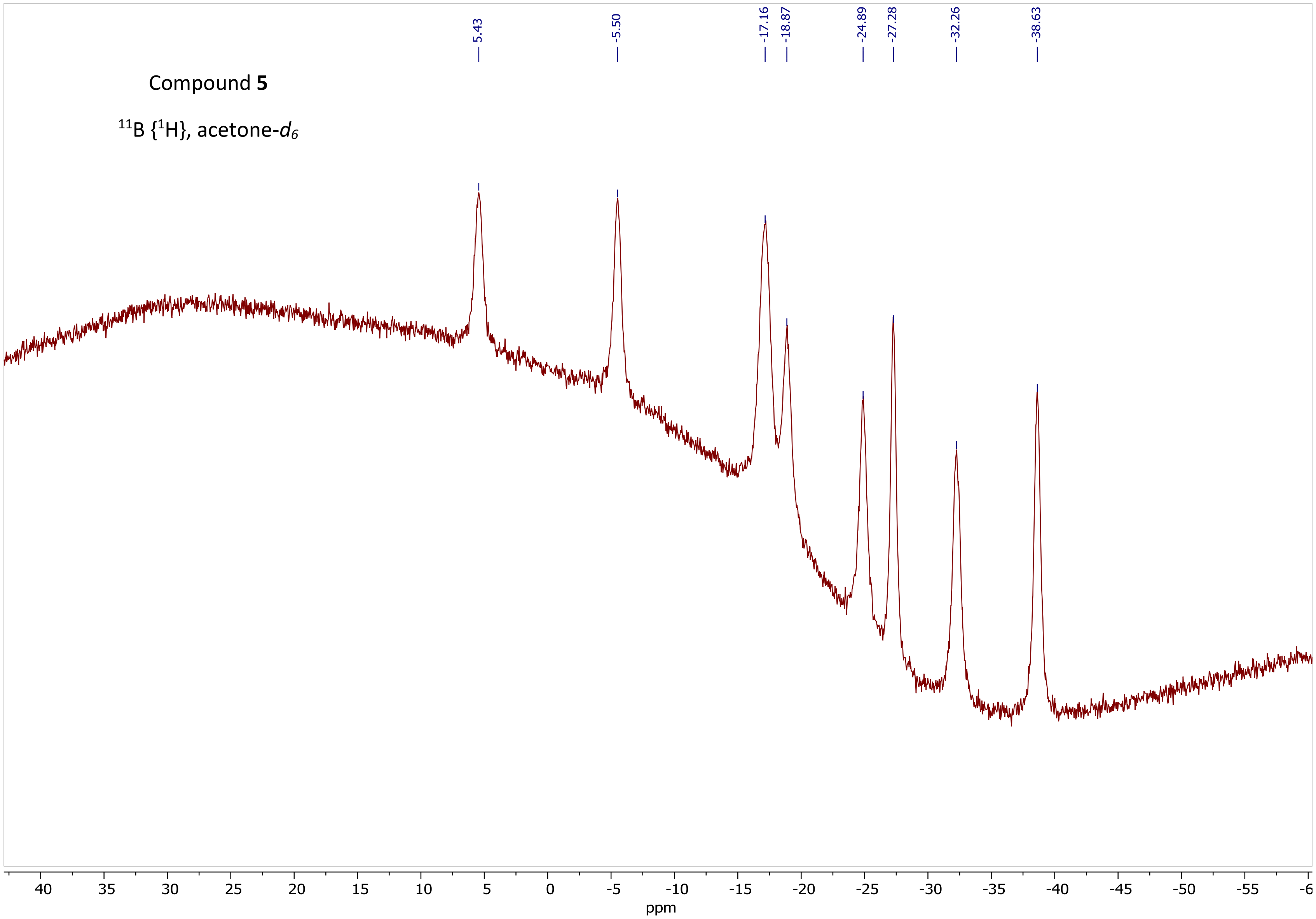

Figure S23. $^{11}B\{^{1}H\}$ NMR spectrum of compound **5**

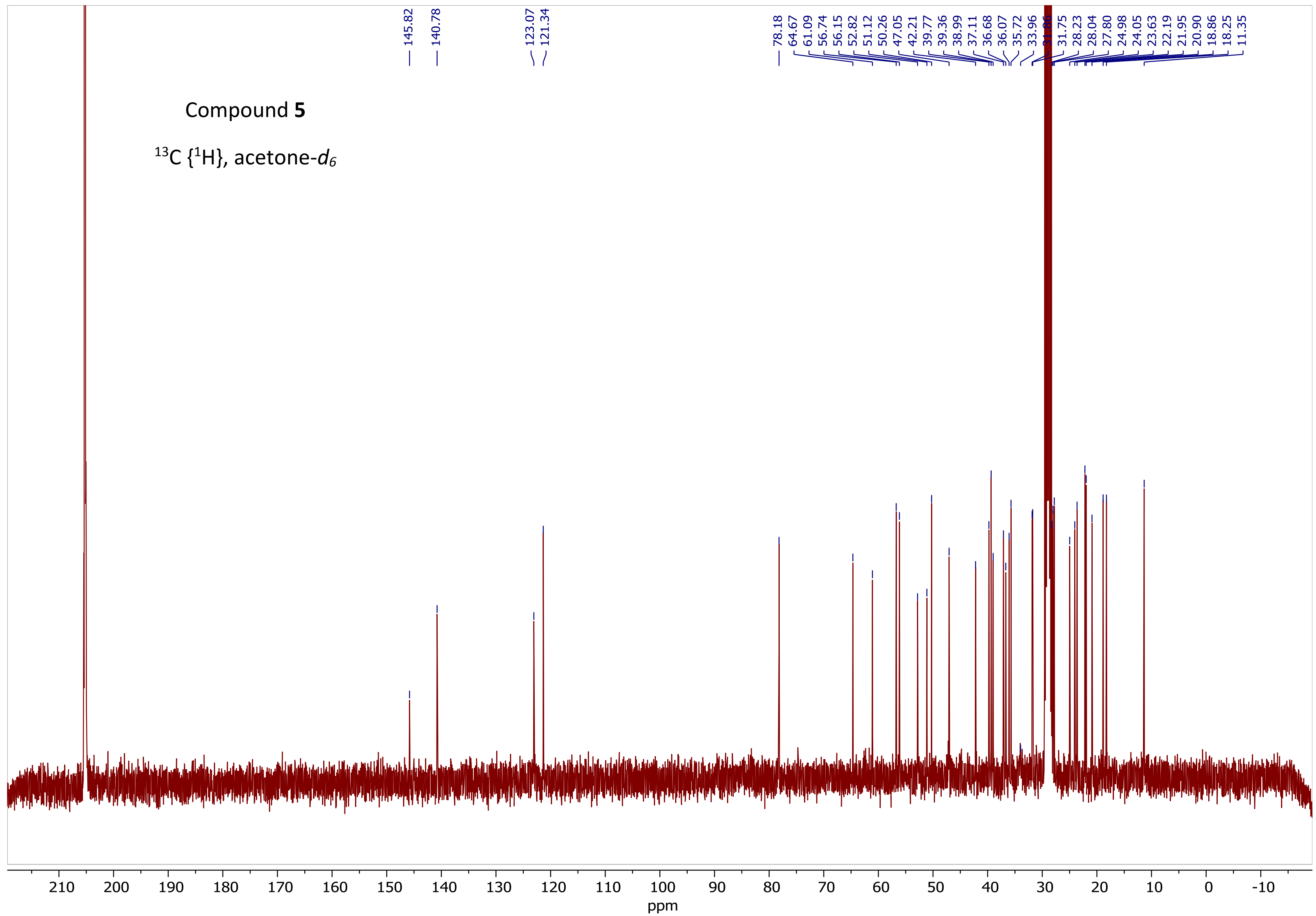

Figure S24. ^{13}C NMR spectrum of compound **5**

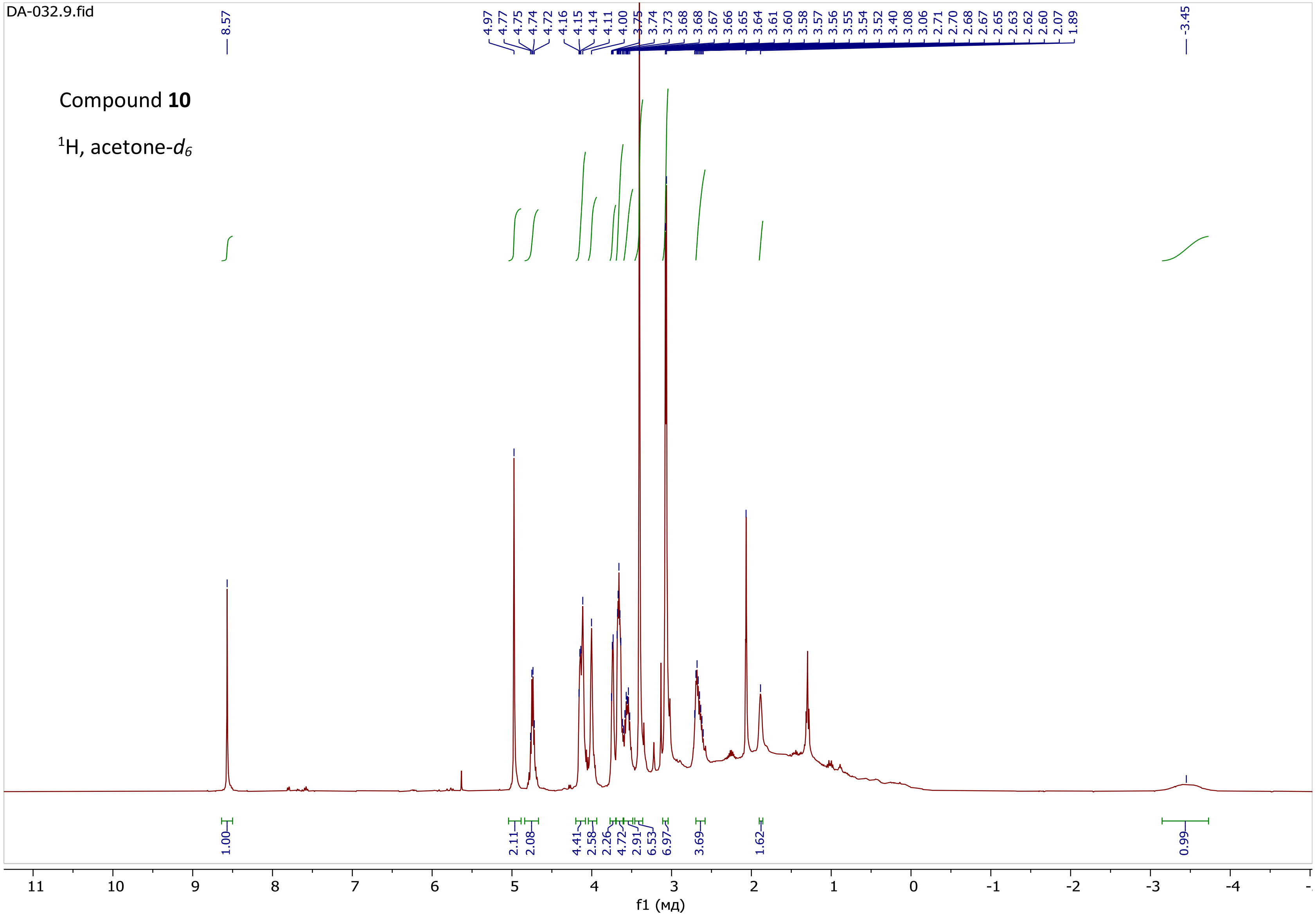

Figure S25. ^{1}H NMR spectrum of compound **10**

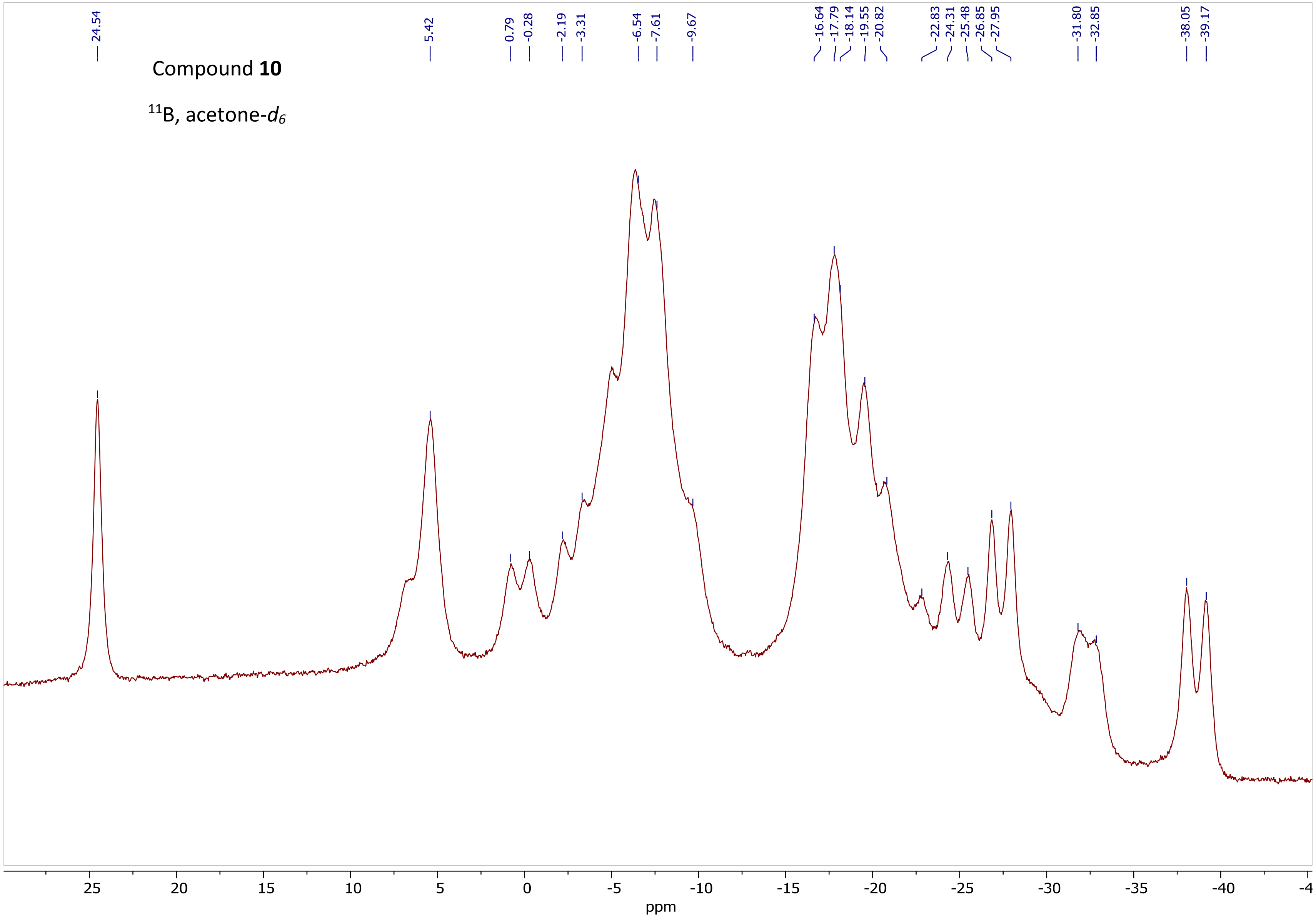

Figure S26. ^{11}B NMR spectrum of compound **10**

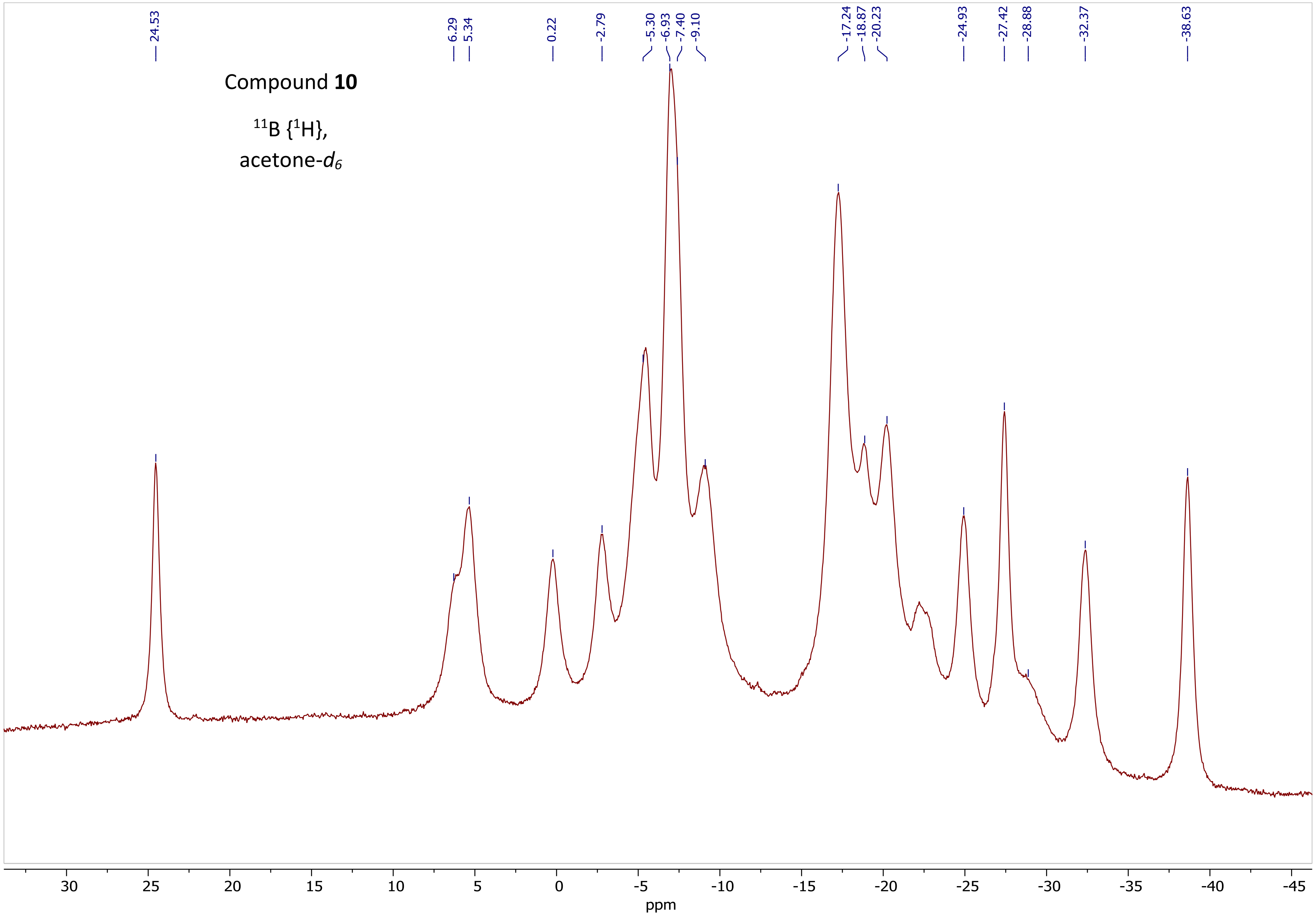

Figure S27. $^{11}B\{^1H\}$ NMR spectrum of compound **10**

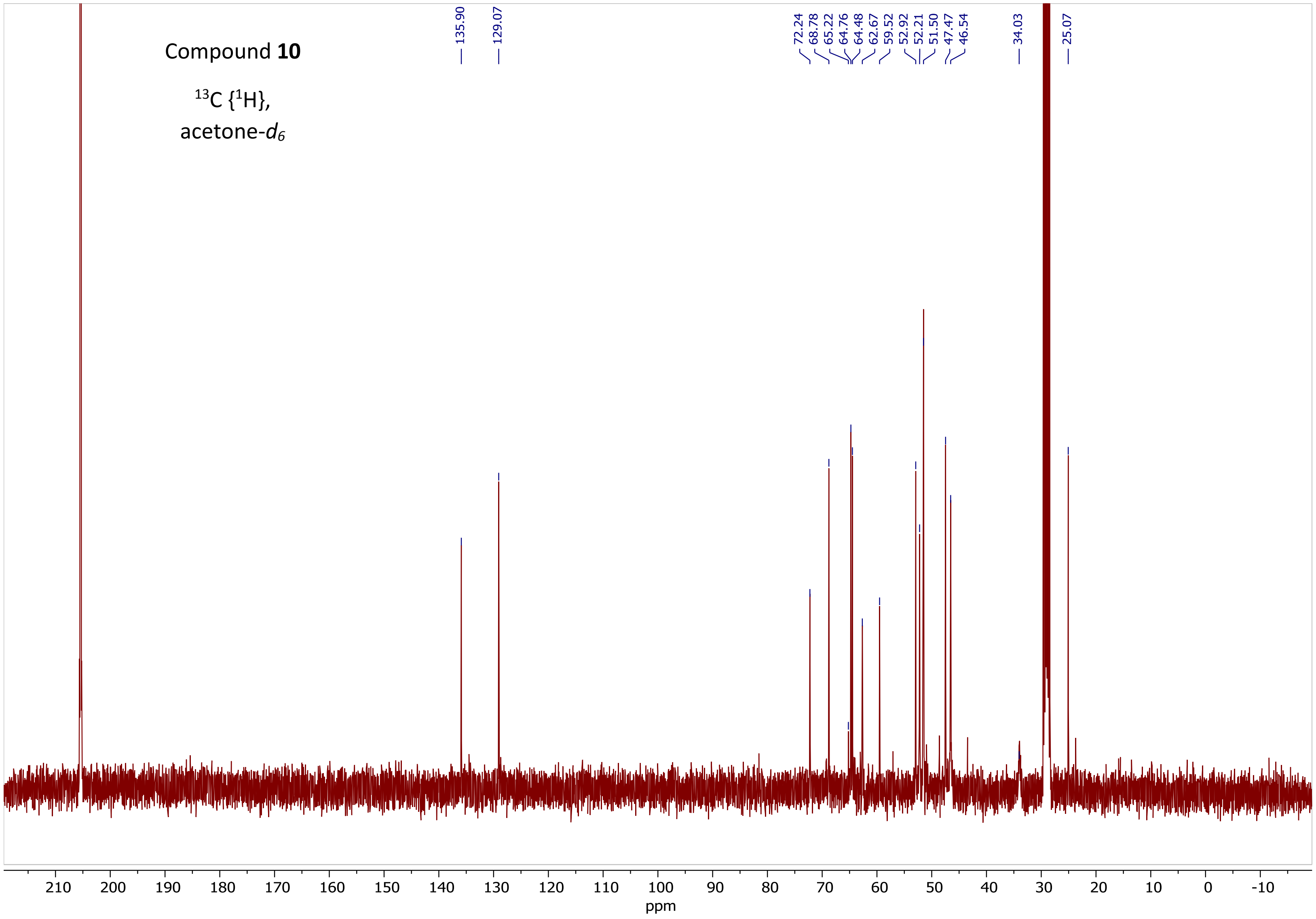

Figure S28. ^{13}C NMR spectrum of compound **10**

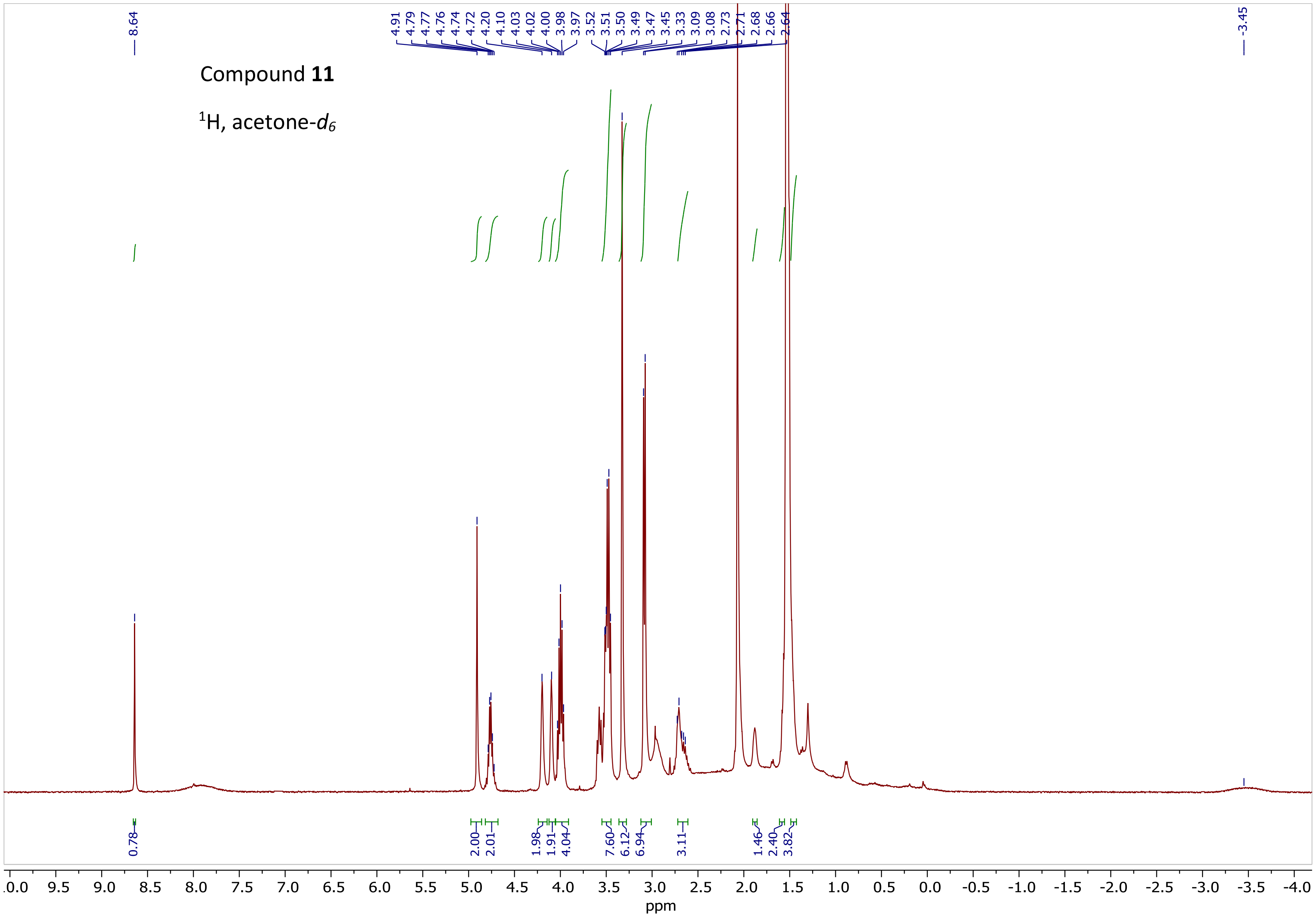

Figure S29. ^{1}H NMR spectrum of compound **11**

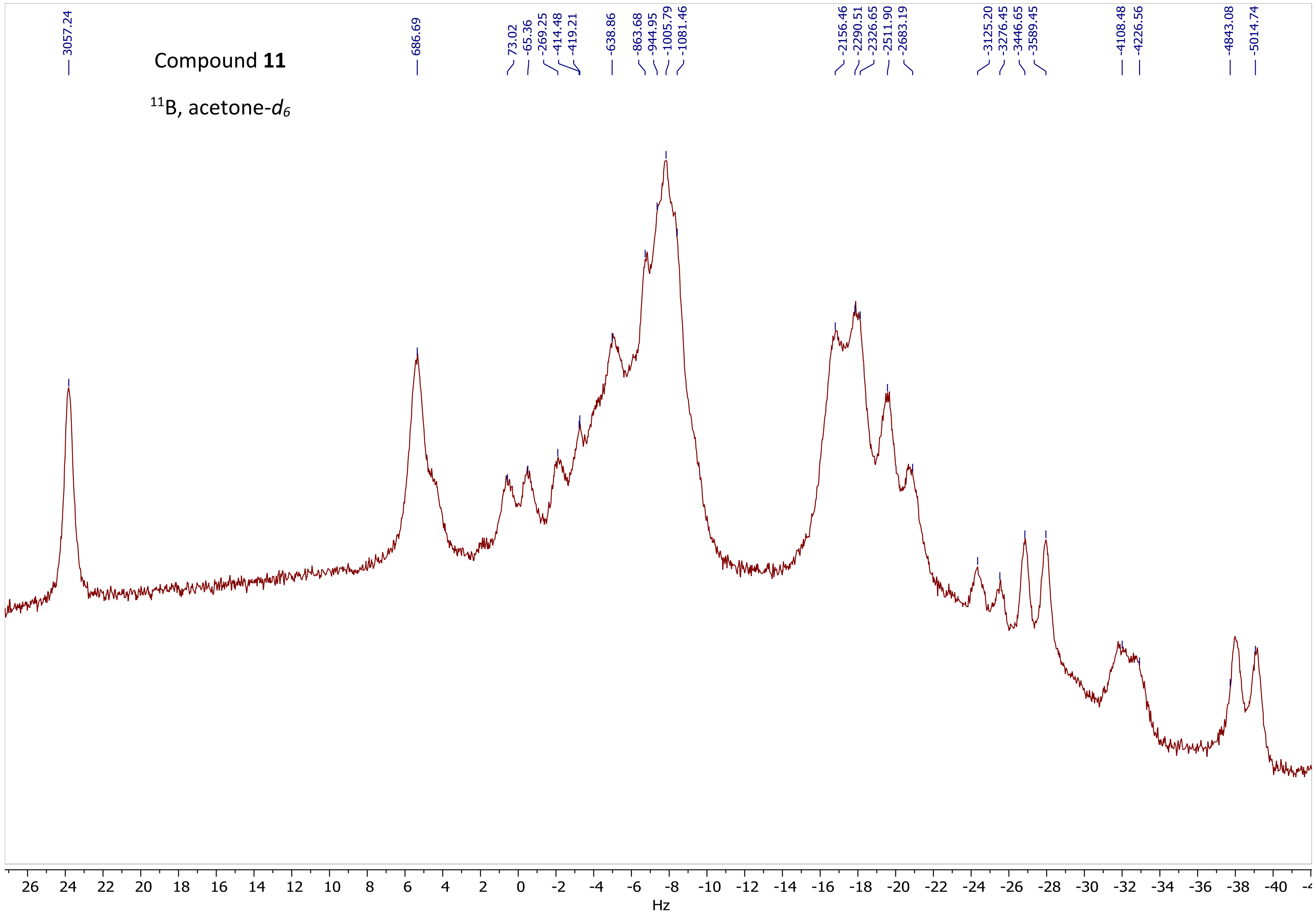

Figure S30. ^{11}B NMR spectrum of compound **11**

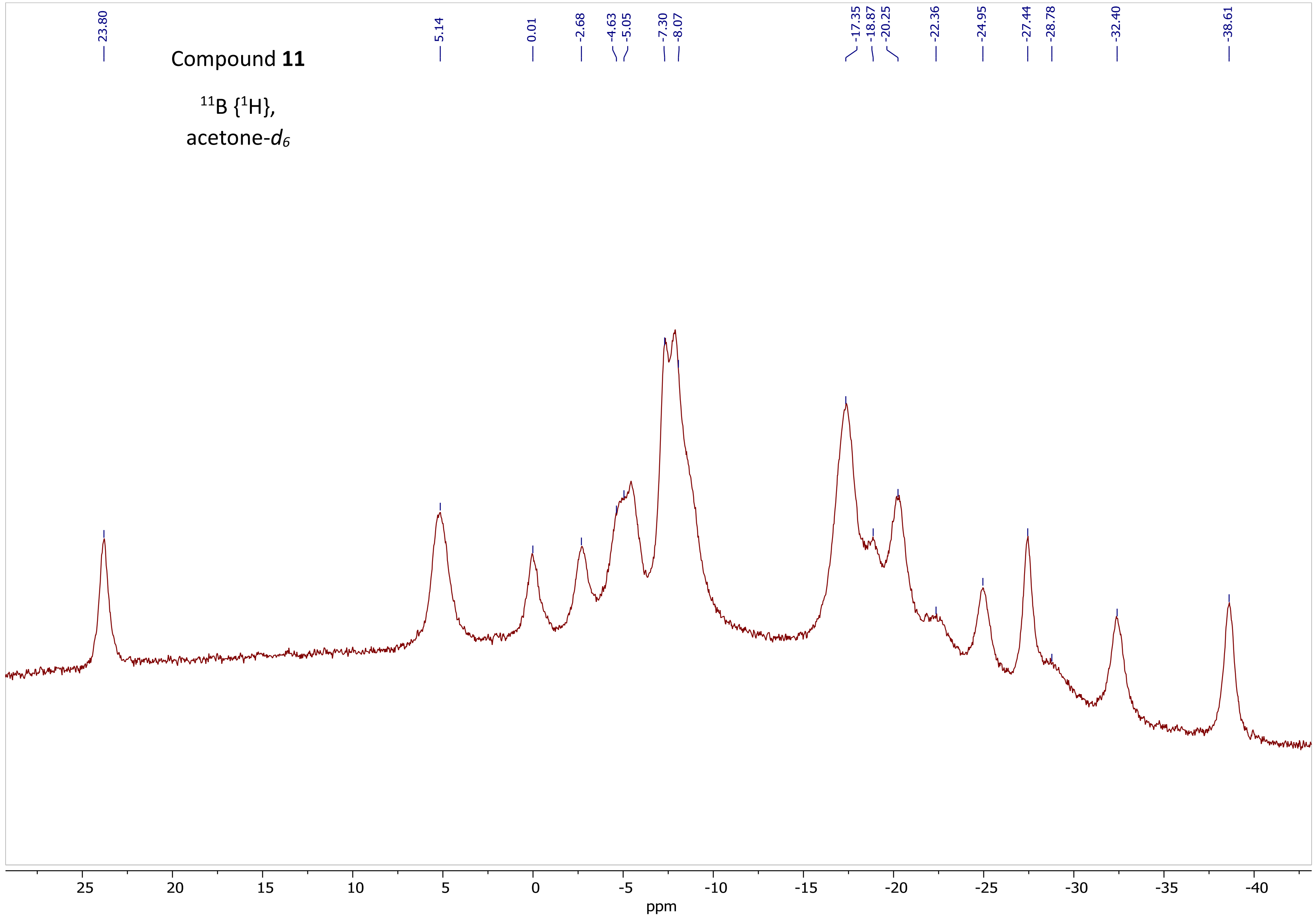

Figure S31. $^{11}B\{^1H\}$ NMR spectrum of compound **11**

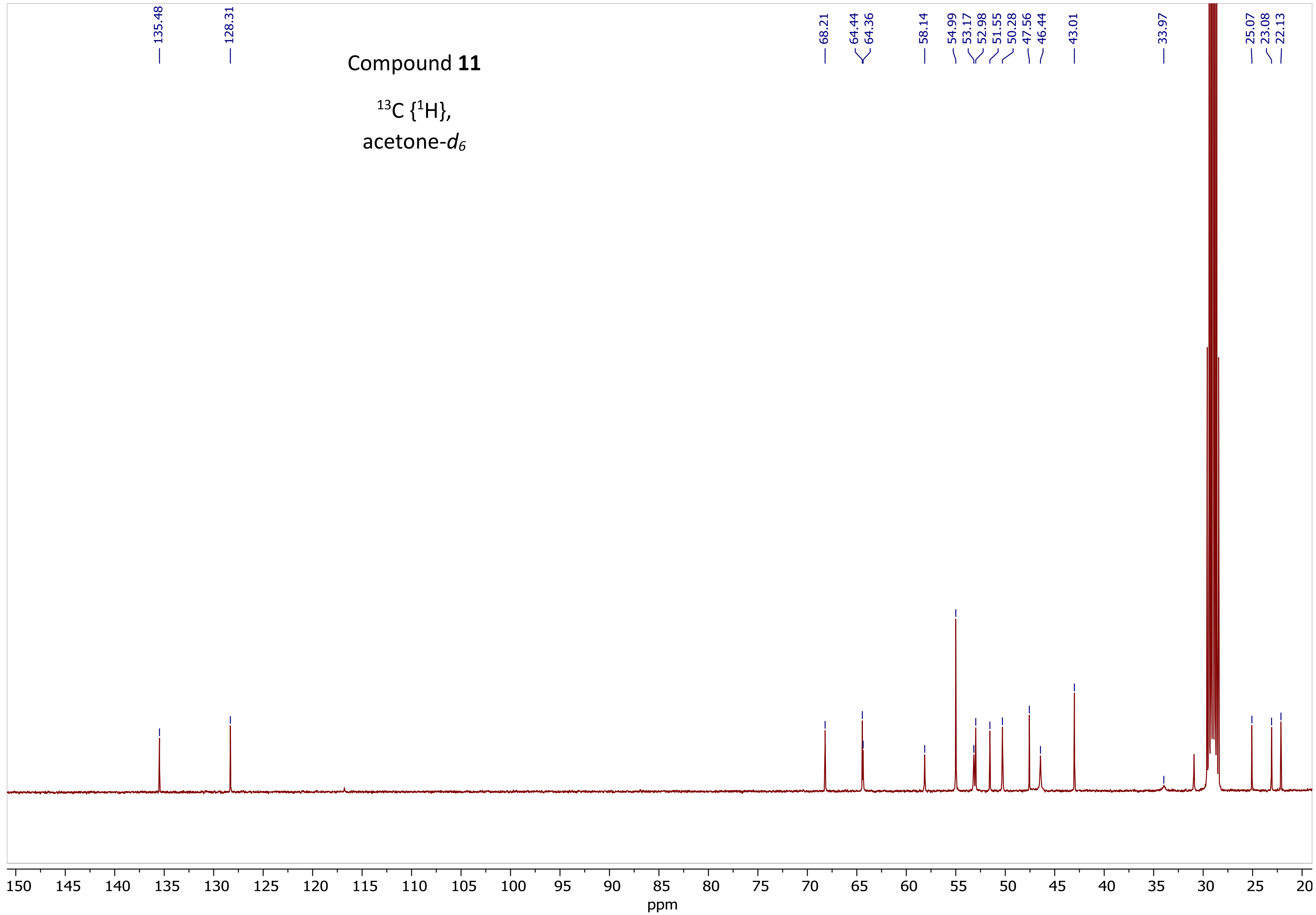

Figure S32. ^{13}C NMR spectrum of compound **11**

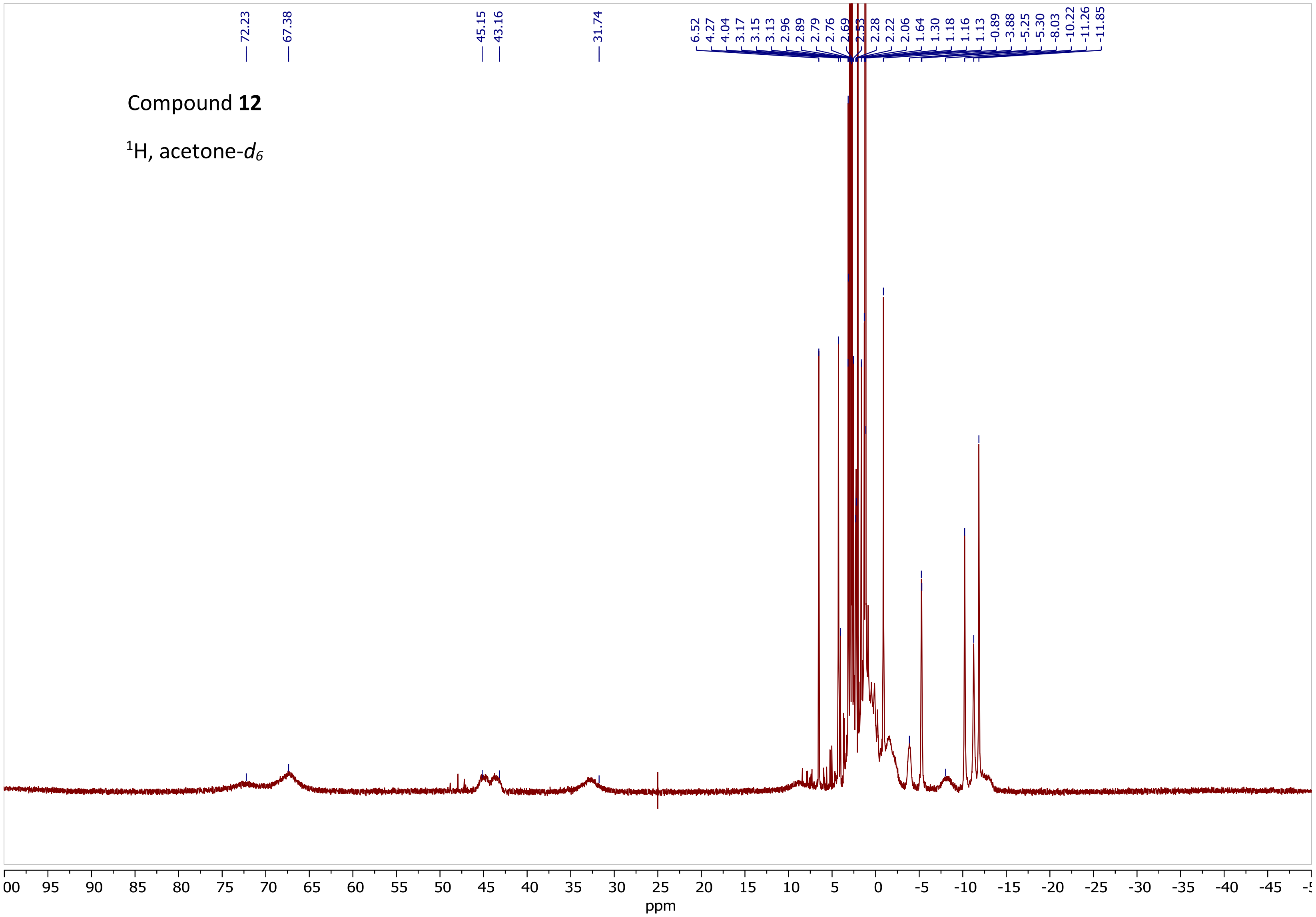

Figure S33. ^{1}H NMR spectrum of compound **12**

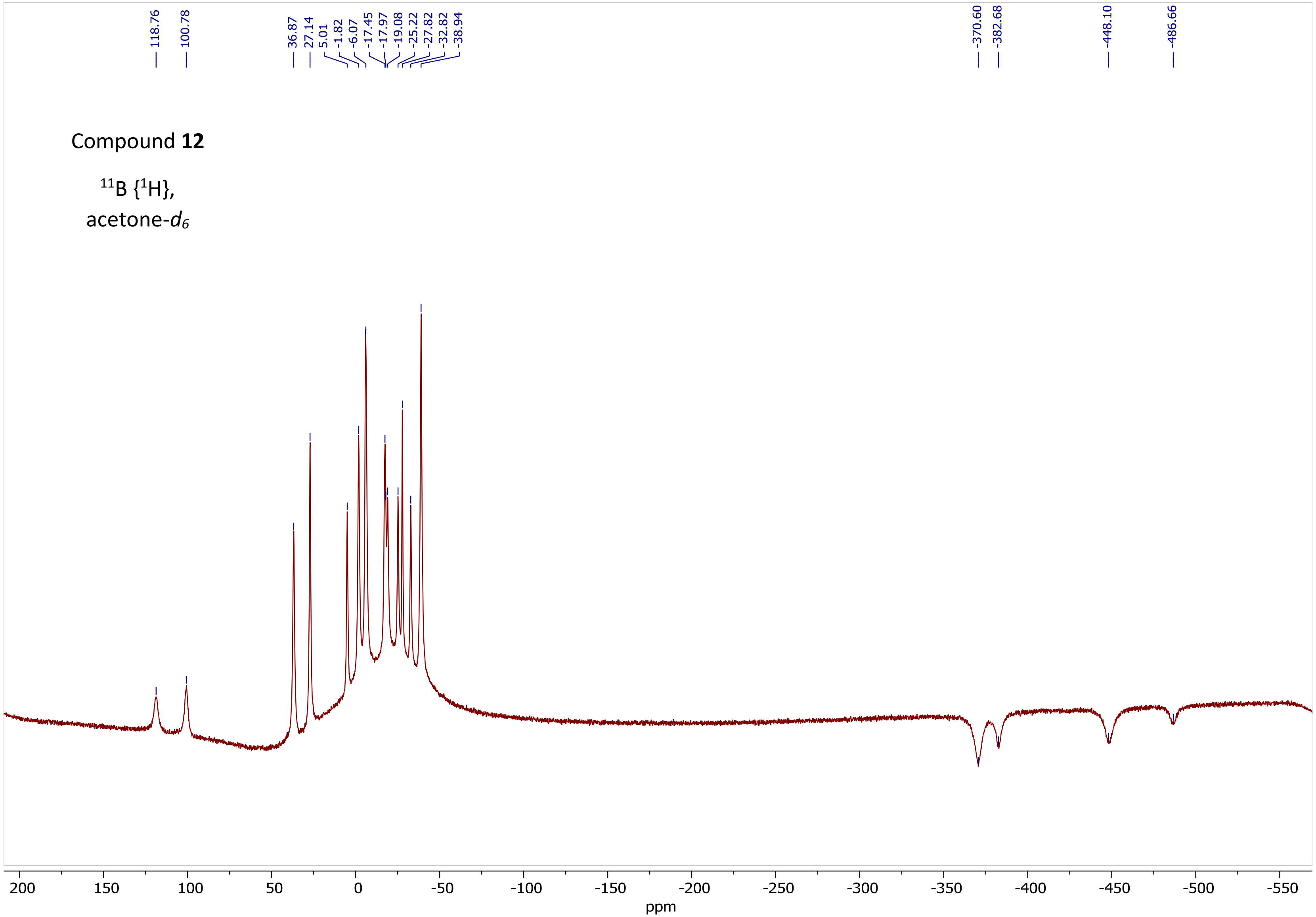

Figure S34. $^{11}B\{^1H\}$ NMR spectrum of compound **12**

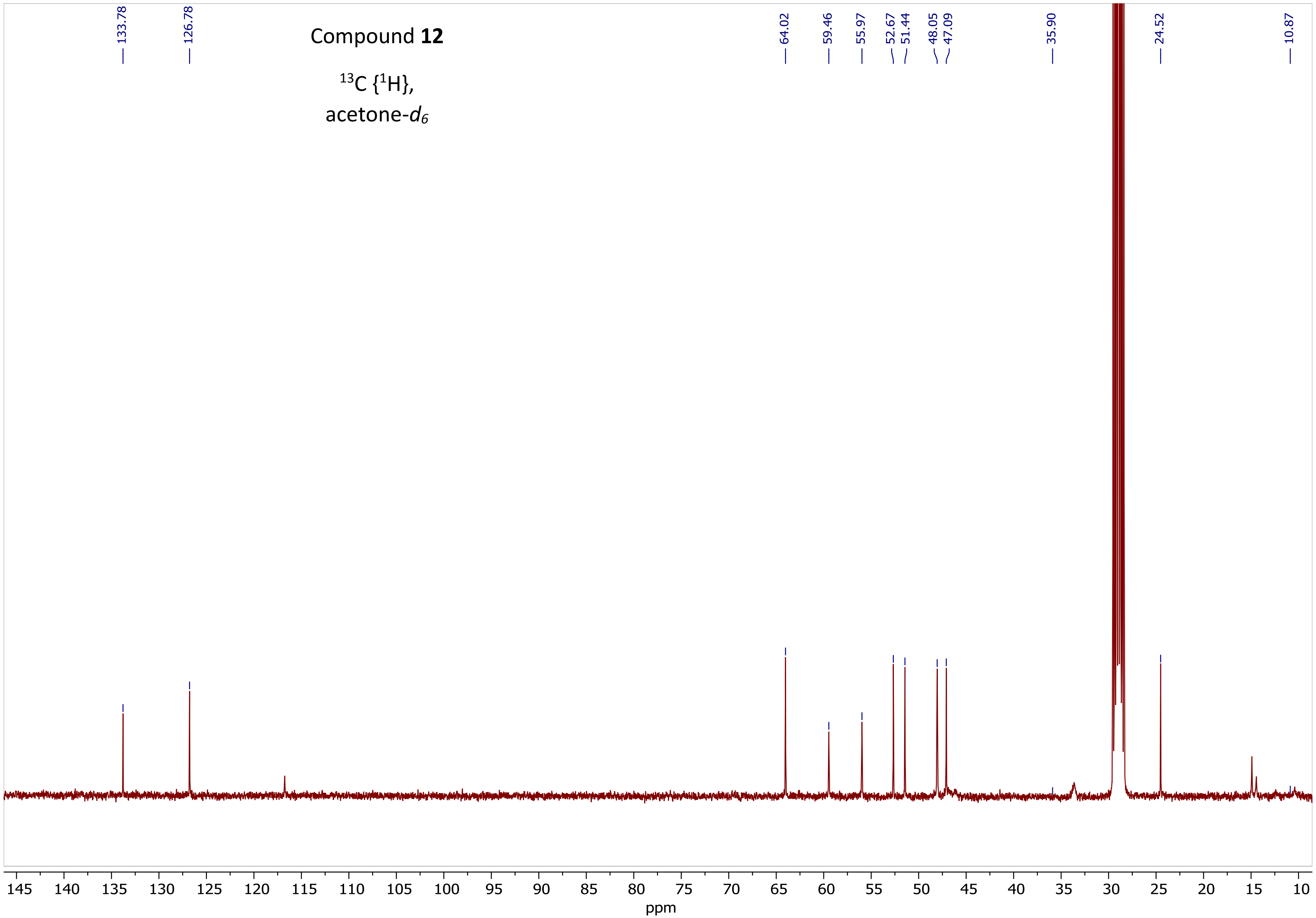

Figure S35. ^{13}C NMR spectrum of compound **12**
